# Trifluoromethylated Flavonoid-Based Isoxazoles as Antidiabetic and Anti-Obesity Agents: Synthesis, In Vitro *α*-Amylase Inhibitory Activity, Molecular Docking and Structure–Activity Relationship Analysis

**DOI:** 10.3390/molecules26175214

**Published:** 2021-08-27

**Authors:** Faisal K. Algethami, Ilyes Saidi, Hani Nasser Abdelhamid, Mohamed R. Elamin, Babiker Y. Abdulkhair, Amani Chrouda, Hichem Ben Jannet

**Affiliations:** 1 Department of Chemistry, College of Science, Imam Mohammad Ibn Saud Islamic University (IMSIU), Riyadh 11432, Saudi Arabia; mohamedrahmt99@gmail.com (M.R.E.); babiker35.by@gmail.com (B.Y.A.); 2 Laboratory of Heterocyclic Chemistry, Natural Products and Reactivity (LR11ES39), Medicinal Chemistry and Natural Products Team, Faculty of Science of Monastir, University of Monastir, Avenue of Environment, Monastir 5019, Tunisia; sisoelyesaidi@live.fr; 3 Department of Chemistry, Advanced Multifunctional Materials Laboratory, Faculty of Science, Assiut University, Assiut 71575, Egypt; chemist.hani@yahoo.com; 4 Department of Chemistry, College of Science Al-Zulfi, Majmaah University, Al-Majmaah 11952, Saudi Arabia; amain.c@mu.edu.sa

**Keywords:** *α*-amylase inhibition, antidiabetic, anti-obesity, cycloaddition, flavonoids, trifluoromethylated flavonoid-based isoxazoles, molecular docking, SAR analysis

## Abstract

Diabetes mellitus is a major health problem globally. The management of carbohydrate digestion provides an alternative treatment. Flavonoids constitute the largest group of polyphenolic compounds, produced by plants widely consumed as food and/or used for therapeutic purposes. As such, isoxazoles have attracted the attention of medicinal chemists by dint of their considerable bioactivity. Thus, the main goal of this work was to discover new hybrid molecules with properties of both flavonoids and isoxazoles in order to control carbohydrate digestion. Moreover, the trifluoromethyl group is a key entity in drug development, due to its strong lipophilicity and metabolic stability. Therefore, the present work describes the condensation of a previously synthesized trifluoromethylated flavonol with different aryl nitrile oxides, affording 13 hybrid molecules indicated as trifluoromethylated flavonoid-based isoxazoles. The structures of the obtained compounds were deduced from by ^1^H NMR, ^13^C NMR, and HRMS analysis. The 15 newly synthesized compounds inhibited the activity of *α*-amylase with an efficacy ranging from 64.5 ± 0.7% to 94.7 ± 1.2% at a concentration of 50 μM, and with IC_50_ values of 12.6 ± 0.2 μM–27.6 ± 1.1 μM. The most effective compounds in terms of efficacy and potency were **3b**, **3h**, **3j**, and **3m**. Among the new trifluoromethylated flavonoid-based isoxazoles, the compound **3b** was the most effective inhibitor of *α*-amylase activity (PI = 94.7 ± 1.2% at 50 μM), with a potency (IC_50_ = 12.6 ± 0.2 μM) similar to that of the positive control acarbose (IC_50_ = 12.4 ± 0.1 μM). The study of the structure–activity relationship based on the molecular docking analysis showed a low binding energy, a correct mode of interaction in the active pocket of the target enzyme, and an ability to interact with the key residues of glycosidic cleavage (GLU-230 and ASP-206), explaining the inhibitory effects of *α*-amylase established by several derivatives.

## 1. Introduction

Obesity and diabetes mellitus constitute major human health problems worldwide [1,2]. Indeed, diabetes mellitus is primarily typified by uncontrolled blood sugar. However, type 1 diabetes mellitus results from disruption in insulin secretion (insulin-dependent), while type 2 usually originates from insulin resistance (alterations of the receptor structure on the target cells, leading to a reduction in hormone–receptor affinity), disruption in insulin secretion, or both [3,4]. Inevitably, inhibiting the degradation of polysaccharides into monosaccharides suitable for absorption into cells implements fundamental strategies to control diabetes and help in the management of obesity [3]. The *α*-amylase enzyme (EC.3.2.1.1) is a digestive enzyme hydrolyzing *α*-1,4-glycosidic bonds of carbohydrates, such as starches [5]. Therefore, *α*-amylase inhibition contributes in one way or another to controlling the digestion of carbohydrates [6,7]; thus, it constitutes a partial pathway to regulate the enzymatic activity of *α*-amylase; hence, it is an ideal therapy for the regulation of obesity and diabetes, as well as their related complications [4]. The majority of commercial *α*-amylase inhibitors—such as acarbose, miglitol, phaseolamine, and voglibose—have been associated with intestinal side effects, such as flatulence, abdominal pain, diarrhea, liver failure, severe skin allergies, and pneumatosis cystoides intestinalis infection [6]. The best *α*-amylase inhibitors are the so-called competitive inhibitors, which inhibit the enzyme irreversibly. A competitive inhibitor binds to the active site of the target enzyme and, thus, prevents the substrate from binding. Therefore, in competitive inhibition, the inhibitor competes with the substrate for the active site. On the other hand, in irreversible inhibition, the inhibitor dissociates very slowly from the target enzyme, as it has become tightly bound to the enzyme. Usually, irreversible inhibitors bind covalently to the active site of the enzyme by modifying the residues or functional groups necessary for enzymatic activity, such that the enzyme is irreversibly inhibited. For a good inhibition of carbohydrate digestion—which can help control obesity, and even type 2 diabetes—we need a non-selective substance capable of inhibiting most of the digestive enzymes of sugars, namely salivary *α*-amylase, pancreatic *α*-amylase, and *α*-glucosidase [8,9].

Previous research on semi-synthesized flavonoids found that isoxazole-based flavonoid derivatives exhibited significant antidiabetic effects by enhancing glucose uptake in insulin-resistant HepG2 cells [10]. Furthermore, flavonoids are the largest group of polyphenolic secondary metabolites, which are commonly found in plants. These secondary metabolites are responsible for the attractive colors of flowers, fruits, and leaves [11]. Structurally, flavonoids have a C_15_ carbon skeleton consisting of two aromatic rings (A) and (B), which are linked together by a C_3_ chain forming the heterocycle (C) [12]. Many biological proprieties have been reported for this type of polyphenolic compound, such as *α*-amylase inhibition [7,13,14,15], antidiabetic [16,17], anticancer [18,19], and antioxidant effects [20], and other biological activities [21,22,23,24,25]. However, this rich biological profile has motivated several chemical research teams to synthesize flavonoids with special moieties and substituents to target particular biological activities [26,27,28,29,30].

On the other hand, isoxazole is a five-membered heterocycle of great importance in medicinal chemistry. Furthermore, naturally occurring isoxazoles—such as ibotenic acid (hallucinogenic, neurotoxin) and muscimol (hallucinogenic, GABA_A_ receptor antagonist) [31,32,33,34]—have attracted increasing attention from chemists and pharmacologists owing to their potent biological and pharmacological properties. Furthermore, isoxazole derivatives are revealed to possess a broad spectrum of biological properties, such as antidiabetic, analgesic, anti-inflammatory, anti-HIV, and anticancer activities [10,35,36,37,38,39,40]. The isoxazole ring has been widely used as a key building block for drugs such as zonisamide (anticonvulsant) [41], valdecoxib and parecoxib (COX-2 inhibitors) [42], and leflunomide (antirheumatic) [43].

In recent decades, the trifluoromethyl group has been in increasing demand for the development of bioactive compounds, due to its strong electron withdrawing character, its metabolic stability, and its lipophilic nature, which are considered important physicochemical properties of bioactive molecules [44]. This fluorinated moiety was found to be indispensable in many important drugs, including fluphenazine (antipsychotic, anticancer) [45], leflunomide (antirheumatic) [43], celecoxib (COX-2 selective inhibitor) [46], fluoxetine (antidepressant) [47], and fluazinam (fungicide) [48]. One review of the literature mentions that trifluoromethylated 5-amino-nicotinic acid has been reported to exhibit promising *α*-amylase activity [49]. In addition, the chlorinated hybrid pyrazole–thiazole molecules were found to be good inhibitors of the *α*-amylase enzyme [50], which also indicates the importance of the chlorine atom in *α*-amylase inhibitory structures.

The above literature data reporting the important physicochemical properties of the trifluoromethyl group—and its contribution, alongside flavonoids and isoxazoles, to inhibiting *α*-amylase and exerting antidiabetic activities—have encouraged us to prepare a new series of hybrid molecules, where a trifluoromethylated flavonoid has been associated with a series of aryl isoxazoles via a methylene linker, and to evaluate their inhibitory power of the *α*-amylase enzyme. The structure–activity relationship (SAR) was studied and approved via molecular docking analysis.

## 2. Results and Discussion

### 2.1. Chemistry

**Scheme 1:**
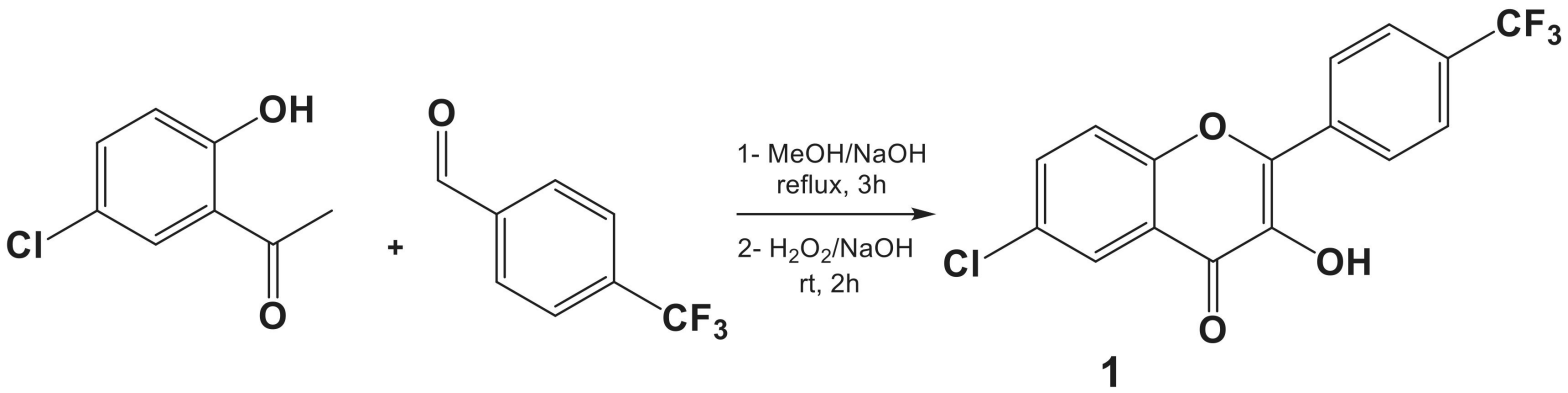
Synthetic pathway of the trifluoromethylated flavonol (**1**).

The preparation of a trifluoromethylated flavonol (**1**)—previously synthesized in our laboratory and showing interesting cytotoxic potential—was reported according to the procedure adopted by Znati et al. (2019) [30]. 5-Chloro-2-hydroxyacetophenone was condensed with 4-(trifluoromethyl)benzaldehyde under reflux of methanol and in the presence of sodium hydroxide for 3 h to produce 5-chloro-4′-(trifluoromethyl)-2-hydroxychalcone; then, the pyranic ring (C) was cyclized and the C-3 carbon was oxidized by the addition of (H_2_O_2_, NaOH) for 2 h at room temperature (Scheme 1). Molecule **1** was obtained with a good yield (79%), after precipitation in ice water.

The structure of flavonol (**1**) was confirmed via spectroscopic analysis and by comparison with literature data [30].

The 1,3-dipolar cycloaddition is a chemical reaction between a 1,3-dipole and a dipolarophile to form a five-membered ring. Hence, 1,3-dipolar cycloaddition is considered the most important way of synthesis of a wide range of isoxazoles. Furthermore, the regiospecific synthesis of isoxazoles catalyzed by Cu(I) under microwave irradiation is based on the [3 + 2] cycloaddition between terminal alkynes (dipolarophiles) and aryl nitrile oxides (dipoles), providing exclusively 3,5-disubstituted regioisomers [39,51].

**Scheme 2:**
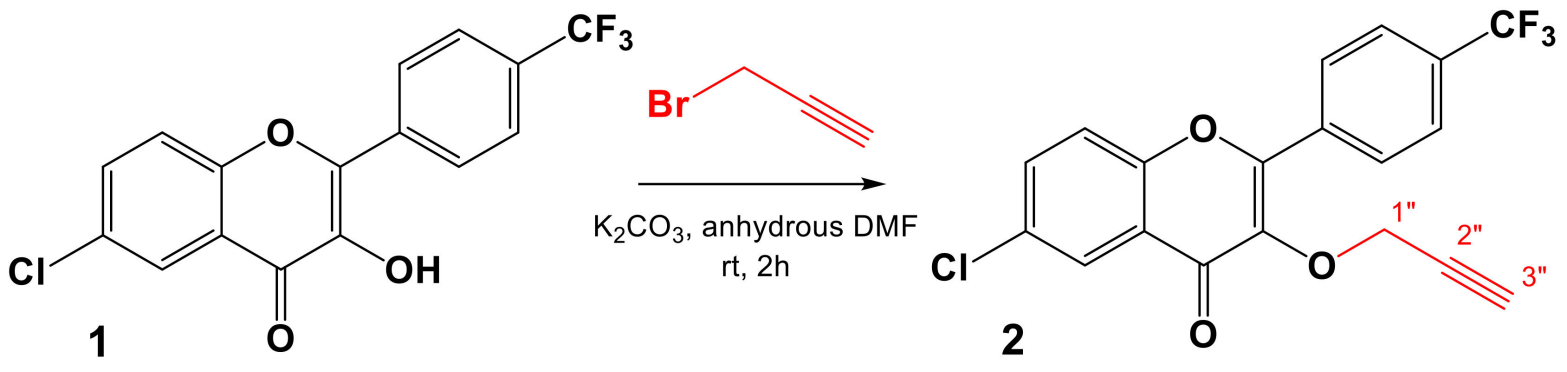
Synthetic pathway of the dipolarophile (**2**).

Our approach to target the trifluoromethylated flavonoid isoxazoles (**3a**–**m**), firstly started by the preparation of the dipolarophile (**2**) via the propargylation of the hydroxyl group in position C-3 of the flavonol (**1**) in anhydrous DMF for 2 h at room temperature, in the presence of K_2_CO_3_, as illustrated in Scheme 2. The dipolarophile (**2**) was prepared with an excellent yield (94%).

The structure of compound **2** was established according to its spectral data. Indeed, in addition to the signals corresponding to the protons and carbons introduced by the flavonol (**1**), new signals of the propargyl moiety were detected in the ^1^H and ^13^C NMR spectra. Moreover, the ^1^H NMR spectrum of the dipolarophile (**2**), recorded in CDCl_3_ at 300 MHz, showed a doublet at δ_H_ 5.06 (2H, d, *J* = 2.4 Hz) corresponding to the methylene proton H-1″ and a triplet at δ_H_ 2.36 (1H, t, *J* = 2.4 Hz) attributable to the proton of the ethynyl group (H-3″). The ^13^C NMR spectrum confirmed the introduction of the propargyl group by the observation of new signals at δ_C_ 78.4, 76.9, and 59.6, attributable to C-2″, C-3″, and C-1″, respectively.

Hydroximyl chlorides **a**–**m** are the main precursors for the in situ generation of aryl nitrile oxides—reagents that participate in the reaction of 1,3-dipolar cycloaddition for the synthesis of isoxazoles. The precursors (**a**–**m**) were synthesized from the appropriate aldehydes according to the general procedure described by Himo et al. (2005) [51]. The desired hydroximyl chlorides (**a**–**m**) were obtained with good yields, ranging from 80 to 98% (Table 1). Thus, the corresponding aryl nitrile oxides were formed in situ by dehydrohalogenation of the corresponding hydroximyl chlorides using the triethylamine as a base.

**Scheme 3:**
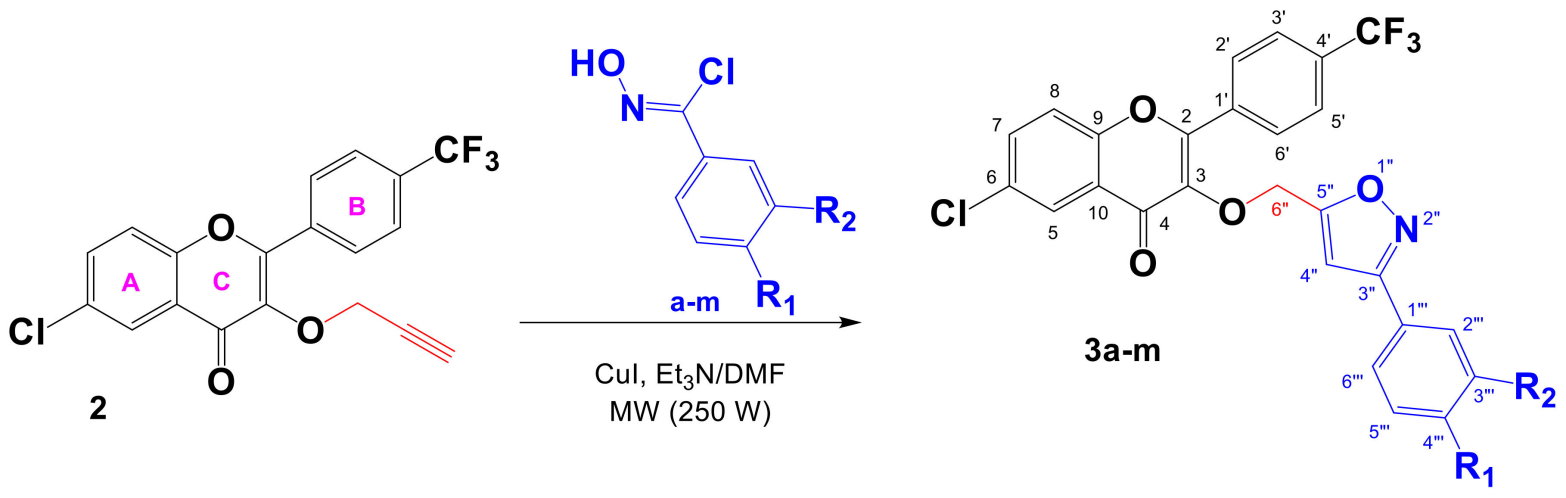
Synthetic pathway of the trifluoromethylated flavonoid isoxazoles **3a**–**m**.

The 1,3-dipolar cycloaddition reaction was applied in a regiospecific approach using the terminal alkyne (**2**) and the different hydroximyl chlorides (**a**–**m**) variously substituted (Scheme 3). The obtained results are listed in Table 1.

All of the reactions were conducted under microwave irradiation (250 W) in DMF in the presence of triethylamine and cuprous iodide (CuI) for 5 min. The products were isolated from the reaction mixture by simple purification on a silica gel column. The newly prepared 3,5-disubstituted isoxazoles (**3a**–**m**) were obtained in yields ranging from 73 to 96% (Table 1).

The structures of the synthesized trifluoromethylated flavonoid-based isoxazoles (**3a**–**m**) (Table 1) were determined by means of ^1^H, ^13^C NMR, and DEPT 135. The ^1^H NMR spectra of these compounds showed a singlet resonating at δ_H_ 6.30–6.60 attributable to the methine proton H-4″ of the isoxazole ring, another singlet at δ_H_ 5.35–5.45 attributable to the methylene proton H-6″, and other signals in the aromatic proton zone relating to the protons introduced by the aryl group. Moreover, these structures were confirmed by their ^13^C NMR and DEPT 135 spectra, showing all of the expected carbon signals—especially the aromatic ones introduced by the used hydroximyl chlorides, as well as the carbon of the methine C-4″ of the isoxazole ring resonating at δ_C_ 102.5–103.0, and that whose signal is inverted in the DEPT 135 spectrum, resonating at δ_C_ 63.5–63.9, attributable to methylene C-6″.

### 2.2. Valuation of α-Amylase Inhibition

To control enzyme activity, inhibition is one of the most successful therapeutic means. Therefore, inhibiting the *α*-amylase enzyme constitutes a partial pathway to manage diabetes and control weight, by hampering the digestion of carbohydrates. To date, drugs such as acarbose have formed part of the arsenal of drugs used to treat DM and obesity. However, acarbose is not used as monotherapy, because of its efficacy and side effects; instead, acarbose is used as add-on therapy, and is very often used in combination with other hypoglycemic drugs. Along with this, this drug is commonly used as a standard compound in the study of *α*-amylase inhibition.

In this study, the 15synthesized compounds (**1**, **2**, and **3a**–**m**) were evaluated for their *α*-amylase inhibitory activity. The results were expressed in potency (IC_50_ ± SEM μM) and efficacy (PI at 50 μM) parameters, and are given in Table 2.

According to the results, the synthesized molecules (**1**, **2**, and **3a**–**m**) possessed significant anti-*α*-amylase activity (*p* ≤ 0.05), with efficacy ranging from 64.5 ± 0.7 to 94.7 ± 1.2% at a concentration of 50 μM, and potencies of IC_50_ values varying from 12.6 ± 0.2 to 27.6 ± 1.1 μM. Among the newly prepared trifluoromethylated flavonoid-based isoxazoles, **3b** (R_1_ = F, R_2_ = H) was found to be the most potent (IC_50_ = 12.6 ± 0.2 μM) and the most effective (PI = 94.7 ± 1.2% at 50 μM) compound to inhibit the activity of the *α*-amylase enzyme. These significant values were found to be comparable to those of acarbose (PI = 97.8 ± 0.5% at 50 μM; IC_50_ = 12.4 ± 0.1 μM), used as a standard substance.

Likewise, compounds **3h**, **3j**, and **3m** can be considered to be bioactive in terms of efficacy and potency (PI = 93.1 ± 0.9%–93.5 ± 1.1% at 50 μM; IC_50_ = 13.3 ± 0.2 μM–13.8 ± 0.1 μM) compared to their analog **3b** and acarbose. The rest of the compounds (**1**, **2**, **3a**, **3c**, **3d**, **3e**, **3f**, **3g**, **3i**, **3k**, and **3l**) were also found to be able to inhibit the *α*-amylase enzyme, but remain less effective (PI = 64.5 ± 0.7%–87.1 ± 0.7% at 50 μM; IC_50_ = 14.4 ± 0.2 μM to 27.6 ± 1.1 μM) than the aforementioned analogs.

The anti-*α*-amylase activity achieved by the synthesized compounds is consistent with previous reports, among which Nie et al. (2020) [10] reported that flavonoid-based isoxazoles could display valuable scaffolds for antidiabetic drug discovery.

By comparing the structures and activities of the synthesized molecules, compounds **3b**–**d**, halogenated in the *para* position of the phenyl group attached to the isoxazole ring, displayed significant efficacy (PI = 85.4 ± 0.9%–94.7 ± 1.2%at 50 μM) and potency (IC_50_ = 12.6 ± 0.2 μM–14.6 ± 0.3 μM) in inhibiting the *α*-amylase enzyme. This finding is in good agreement with the literature, showing that the presence of halogen atoms (F, Cl, or Br) in the structure was essential for a potent *α*-amylase inhibition [49,50]. In addition, the structure–activity relationship study allowed us to conclude that more inductive attractor (−I) and mesomeric donor (+M) effects increase the *α*-amylase inhibitory efficacy and potency (activity of the fluorinated derivative **3b** (R_1_ = F, R_2_ = H; PI = 94.7 ± 1.2% at 50 μM; IC_50_ = 12.6 ± 0.2 μM) is higher than that of the chlorinated derivative **3c** (R_1_ = Cl, R_2_ = H; PI = 87.1 ± 0.7% at 50 μM; IC_50_ = 14.4 ± 0.2 μM), followed by the brominated derivative **3d** (R_1_ = Br, R_2_ = H; PI = 85.4 ± 0.9% at 50 μM; IC_50_ = 14.6 ± 0.3 μM)), in accordance with previous studies [4,49]. It is evident that the *α*-amylase inhibition increases with the inductive attractor (−I) and mesomeric donor (+M) effects of halogen atoms.

The compound **3h** (R_1_ = OMe, R_2_ = H), bearing a *para*-methoxy group, exhibited interesting *α*-amylase inhibitory activity, with an efficacy of 93.5 ± 1.1% at 50 μM and an IC_50_ value of 13.3 ± 0.2 μM. This finding is consistent with previous results [49]. Moreover, the compound **3i** (R_1_ = O-*n*-Bu, R_2_ = H; IC_50_ = 15.6 ± 0.2 μM; PI = 79.4 ± 1.0% at 50 μM), bearing a *para*-substituted butoxy group, perceived a lower activity than that of the *para*-methoxylated compound **3h**. The electronic effects of alkoxyl groups (−I and +M), comparable to those of halogens, may explain their significant activities. Moreover, the methoxylation or the halogenation in the *meta* position of the *para*-alkoxylated compounds (**3h** and **3i**), producing the compounds **3j**–**m** (IC_50_ = 13.7 ± 0.2 μM–23.0 ± 0.2 μM), did not show a well-defined effect; they reduced the enzyme activity of the α-amylase in some cases and increased it in others, but **3h** remains the most potent and effective alkoxylated derivative.

Likewise, it should be noted that the compound **3g** (R_1_ = NO_2_, R_2_ = H), bearing a *para*-nitro group, revealed sufficient efficacy (PI = 74.7 ± 0.3%at 50 μM) and potency (IC_50_ = 18.1 ± 0.3 μM) to inhibit the *α*-amylase enzyme, consistent with the results of Nawaz et al. (2020) [49]. The activity of this molecule may be due to the inductive attracting effect (−I) of the nitro group.

Conversely, unsubstituted (**3a**: R_1_ = H, R_2_ = H; PI = 65.6 ± 0.2% at 50 μM; IC_50_ = 27.2 ± 0.7 μM), *para*-methylated (**3e**: R_1_ = CH_3_, R_2_ = H; PI = 65.4 ± 0.4% at 50 μM; IC_50_ = 26.0 ± 0.7 μM), and *para*-*tert*-butylated (**3f**: R_1_ = *tert*-Bu, R_2_ = H; PI = 64.5 ± 0.7% at 50 μM; IC_50_ = 27.6 ± 1.1 μM) compounds did not demonstrate remarkable *α*-amylase inhibition activity compared to their analogs. This finding shows that the increase in the donor inductive effect (+I) does not have a practically remarkable effect on the inhibitory activity of the *α*-amylase enzyme. This result is consistent with previously reported data [4,49].

### 2.3. Molecular Docking Studies

The *α*-amylase enzyme (EC 3.2.1.1)—a glycosyl hydrolase—hydrolyzes *α*-1,4-glycosidic bonds in starches, such as amylose. The crystal structure of the *α*-amylase enzyme (PDB: 7TAA) is composed of a chain A with a sequence length of 478 amino acids [52]. *α*-Amylase is one of the most important key enzymes responsible for carbohydrate digestion [5]; thus, blocking the *α*-amylase enzyme offers a strategy to control diabetes and help in the management of obesity [6,7]. Extensive molecular docking analysis was executed to explore the binding mode, determine the likely interactions of synthesized molecules (**1**, **2**, and **3a**–**m**) within the hydrophobic binding pocket of ABC (modified acarbose hexasaccharide) of the protein crystal structure of *Aspergillus oryzae α*-amylase enzyme (PDB: 7TAA) [52] using AutoDock Vina software, and rationalize the observed in vitro *α*-amylase inhibitory activity of the newly synthesized compounds.

All of the synthesized molecules (**1**, **2**, and **3a**–**m**) —and acarbose, used as a standard substance—were studied *in silico*. Binding energies and interaction details (number of interactions, number of interacting amino acids, interacting amino acids and hydrogen bonds) of ligands with the target enzyme are presented in Table 2.

From the in silico docking results (Table 2), it was observed that compounds **1** and **2** exhibited good binding energies (−7.7 and−7.8 kcal/mol, respectively) within the binding site of the *α*-amylase enzyme (PDB: 7TAA). These values are comparable to that of the standard substance (acarbose, −7.9 kcal/mol), allowing them to fit favorably into the active site of the target enzyme. Interestingly, the newly synthesized trifluoromethylated flavonoid-based isoxazoles (**3a**–**m**) gave the expected results; they were properly in the bonding pose, and exhibited magnificent binding energies ranging from −9.6 to −8.2 kcal/mol (Table 2). This would allow all of them to fit favorably into the active site of the *α*-amylase enzyme, better than acarbose.

The binding mode of the three most active compounds (**3b**, **3h**, and **3j**) with the target enzyme are discussed below.

**Figure 1:**
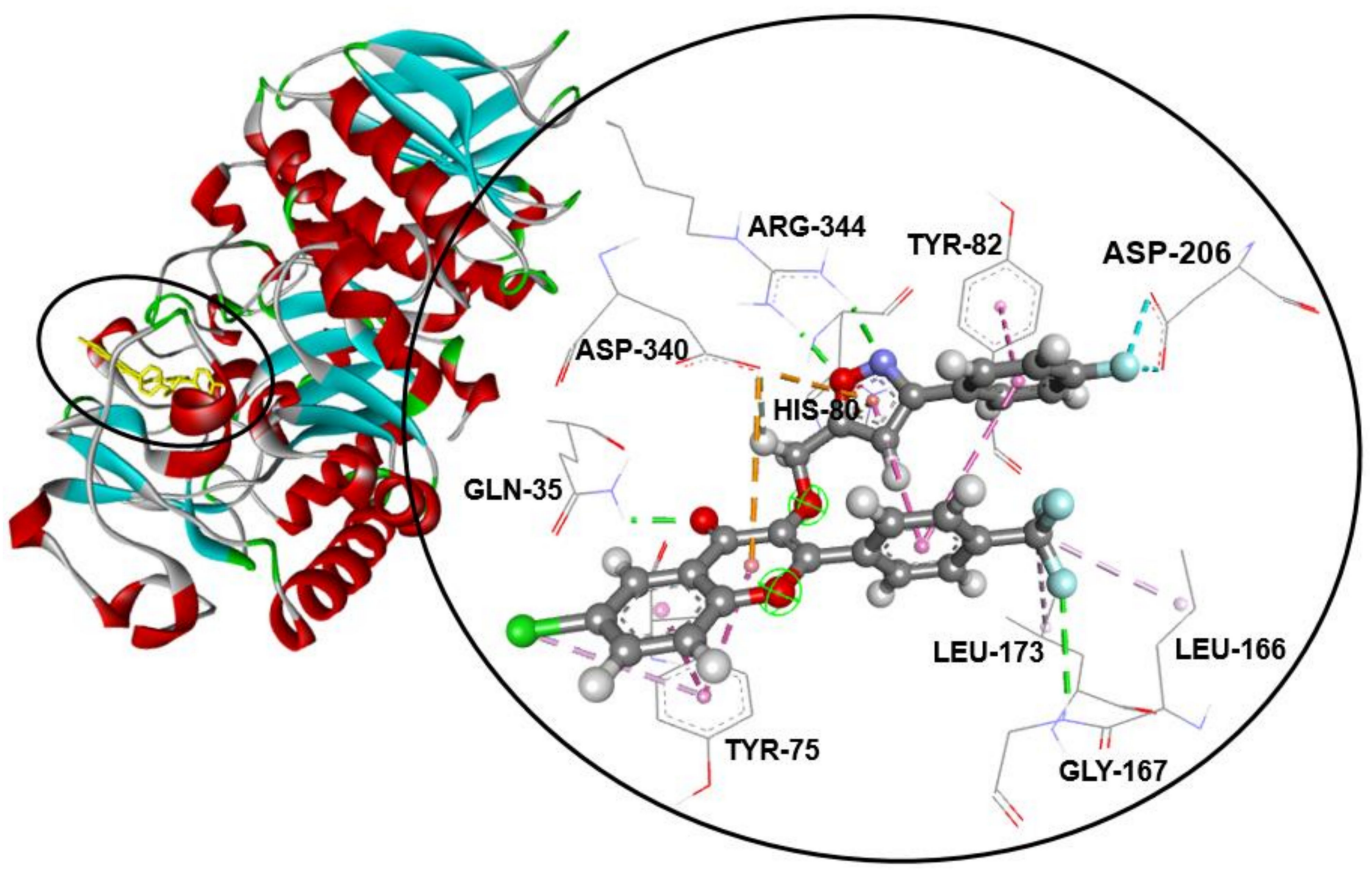
Compound **3b** fits into the hydrophobic binding pocket of ABC in PDB: 7TAA.

Compound **3b**—the most potent and effective inhibitor of the *α*-amylase enzyme in the tested series of trifluoromethylated flavonoid-based isoxazoles—exhibited an excellent binding energy of −9.6 kcal/mol. In addition, the binding mode of **3b** suggests that it is implicated in 16non-covalent interactions with 10amino acids (Table 2). Thoroughly, the 3-(3-(4-fluorophenyl)isoxazol-5-yl)methoxyl moiety forms two halogen (fluorine) bonds with the ASP-206, a Pi–Pi T-shaped bond with the TYR-82, a Pi–cation bond with the HIS-80, two conventional hydrogen bonds with ARG-344, and a Pi–anion and a carbon–hydrogen bond with ASP-340 residue. Furthermore, the trifluoromethyl group attached to C-4′ of the flavonoid skeleton is involved in conventional hydrogen binding with GLY-167 and two alkyl bonds with the LEU-166 and LEU-173 amino acids. On the other hand, the flavonoid skeleton interacts via a Pi–anion bond with ASP-340, a conventional hydrogen bond with GLN-35, and a Pi–alkyl and two Pi–Pi stacked bonds with TYR-75 (Figure 1).

**Figure 2:**
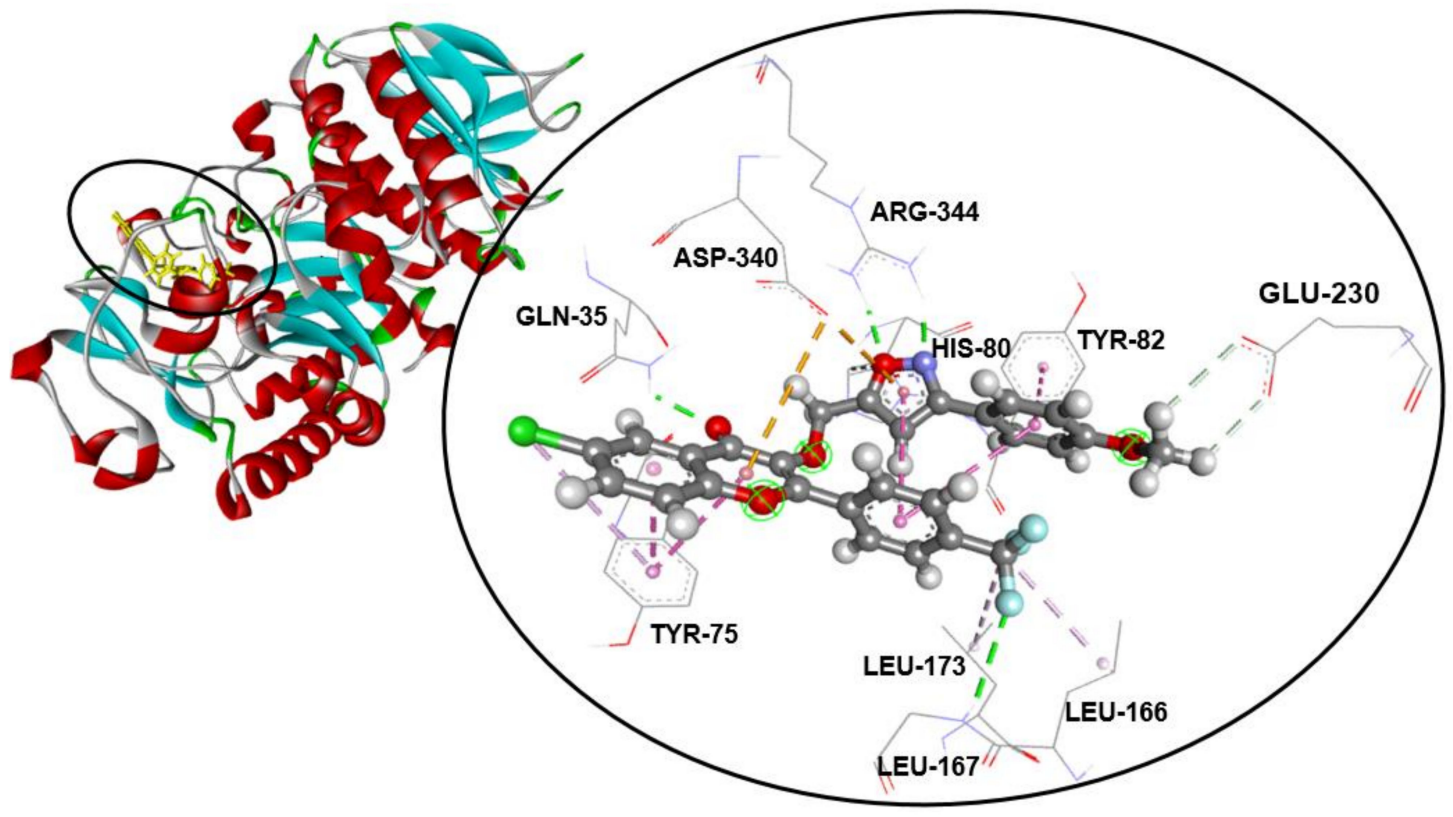
Compound **3h** fits into the hydrophobic binding pocket of ABC in PDB: 7TAA.

Furthermore, compound **3h** exhibited an interesting binding energy of −9.3 kcal/mol. Its binding mode shows that it is involved in 15 non-covalent interactions with 10 residues (Table 2). Accordingly, molecular insight from docking analysis suggests that the 3-(3-(4-methoxyphenyl)isoxazol-5-yl)methoxyl moiety forms two carbon–hydrogen bonds with GLU-230, a Pi–Pi T-shaped bond with TYR-82, two conventional hydrogen bonds with ARG-344, a carbon–hydrogen bond with HIS-80, and a Pi–anion bond with the ASP-340 residue. Moreover, the trifluoromethyl group is involved in a conventional hydrogen bond with GLY-167, and two alkyl interactions with the LEU-166 and LEU-173 amino acids. In addition, the flavonoid skeleton is involved in a conventional hydrogen bond with GLN-35, a Pi–anion bond with ASP-340, and two Pi–Pi stacked bonds and a Pi–alkyl bond with TYR-75 (Figure 2).

**Figure 3:**
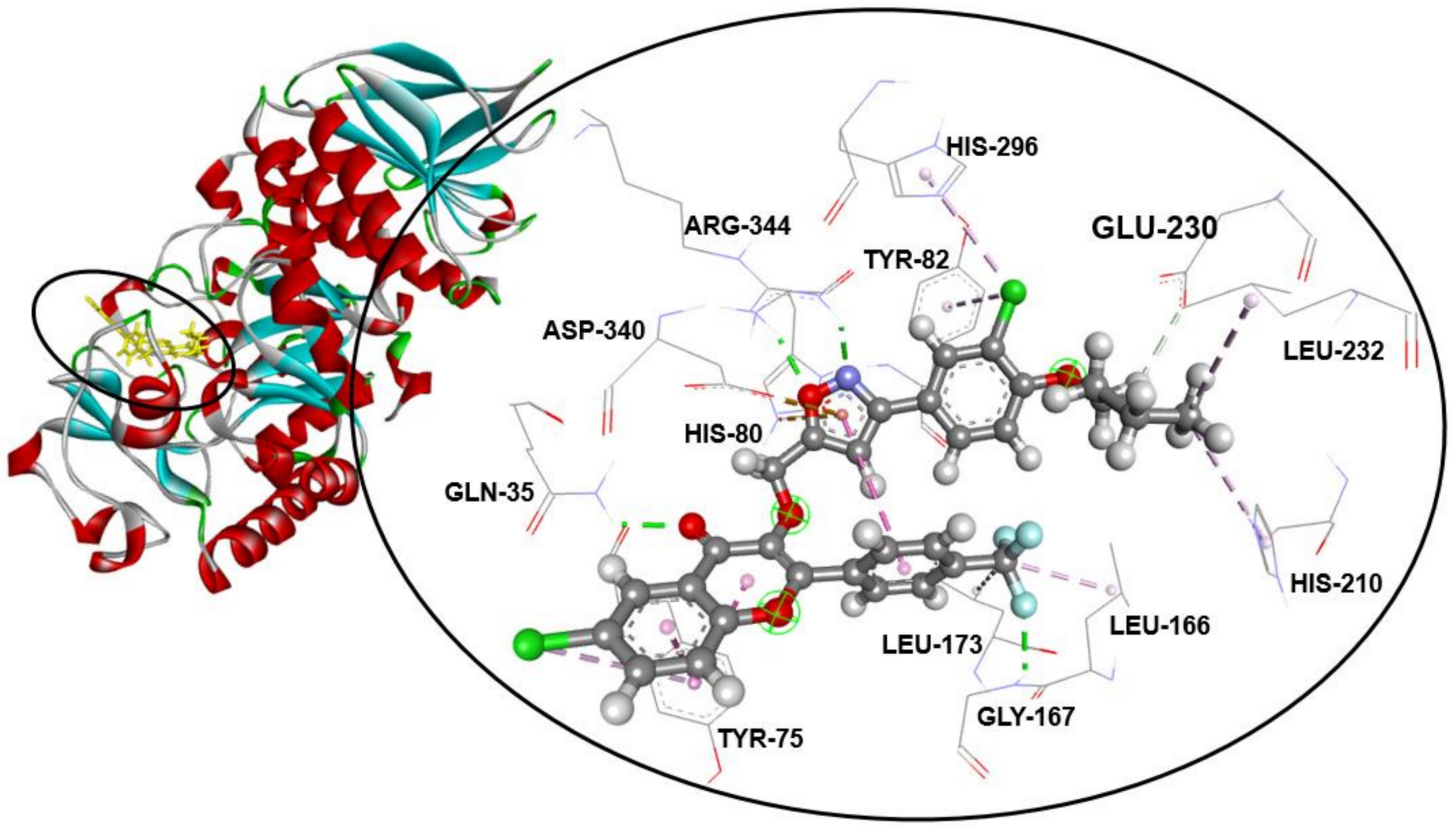
Compound **3j** fits into the hydrophobic binding pocket of ABC in PDB: 7TAA.

On the other hand, compound **3j** exhibited a significant binding energy of −9.2 kcal/mol. Its binding mode shows that it is implicated in 16non-covalent interactions with 13amino acids (Table 2). Therefore, in-depth analysis of molecular docking suggests that the 3-(3-(4-butoxy-3-chlorophenyl)isoxazol-5-yl)methoxyl moiety forms two conventional hydrogen bonds with ARG-344, three Pi–alkyl bonds with TYR-82, HIS-210, and HIS-296, an alkyl interaction with LEU-232, a carbon–hydrogen bond with the GLU-230 residue, a Pi–anion bond with ASP-340, and a Pi–cation bond with HIS-80. Additionally, the trifluoromethyl group linked to the aromatic ring B of the flavonoid skeleton is involved in a conventional hydrogen bond with GLY-167 and two alkyl interactions with the LEU-166 and LEU-173 residues. Moreover, the flavonoid skeleton is involved in a conventional hydrogen bond with GLN-35 and a Pi–alkyl and two Pi–Pi stacked bonds with the amino acid TYR-75 (Figure 3).

In the *Aspergillus oryzae α*-amylase enzyme (PDB: 7TAA), the nucleophile and the catalytic acid/base residues, which are responsible for the cleavage of glycosidic bonds, are the ASP-206 and GLU-230 amino acids, respectively [5]. Therefore, interactions between protein and ligand, primarily via the GLU-230 and ASP-206 residues, are the best way to block the cleavage of glycosidic bonds. In fact, the low binding energy, the correct binding pose, the ability to interact with key residues of glycosidic cleavage (GLU-230 and ASP-206), and the large number of interactions in the active pocket of the target enzyme (Table 2) can explain the strong inhibitory *α*-amylase efficacy established by most compounds.

## 3. Materials and Methods

### 3.1. General Experimental Procedures

All used solvents were freshly distilled prior to use. All chemical reactions were monitored using commercial TLC plates (Silica gel 60, F_254_, sds). A Büchi 510 apparatus was used to determine melting points. All ^1^H NMR (300 MHz), DEPT 135, and ^13^C NMR (75 MHz) spectra were recorded in deuterated chloroform and dimethyl sulfoxide-*d*_6_ with a Bruker AC-300 spectrometer. All chemical shifts (δ) were reported in parts per million (ppm), coupling constants (*J*) were given in Hertz (Hz), and residual non-deuterated solvent resonance was used as an internal reference. DCI-HRMS spectra were run in a Waters GCT 1er in the positive ion mode. ESI-HRMS spectra were recorded with ESI-TOF (Waters LCT, Markham, Ontario, Canada) in the positive ion mode using the reflectron mode.

### 3.2. Chemistry

#### 3.2.1. General Procedure for the Synthesis of the Flavonol **1**

First, 2.89 g of 4-(trifluoromethyl)benzaldehyde (16.61 mmol), 5-chloro-2-hydroxyacetophenone (2 mL, 16.61 mmol), and 2 g of sodium hydroxide (50 mmol) were added to 100 mL of methanol in a round-bottomed flask (500 mL) placed on a magnetic stirrer. The mixture was refluxed for 3 h. The reaction mixture was cooled for 15 min to room temperature. Subsequently, 100 mL of sodium hydroxide (0.5 M), and then 6.84 mL of hydrogen peroxide (35%), were added, and the mixture was stirred at room temperature for 3 h. The reaction mixture was poured into ice water to produce a precipitate, which was then recovered via filtration to target compound **1** [30]. The trifluoromethylated flavonol (**1**) was obtained with a yield of 79%.

##### 6-Chloro-3-hydroxy-2-(4-(trifluoromethyl)phenyl)-4*H*-chromen-4-one (**1**) 

Red solid, yield: 79%, mp: 275 °C, ^1^H NMR (DMSO-*d_6_*, 300 MHz) δ_H_ 7.95 (1H, d, *J* = 2.4 Hz, H-5), 7.57 (1H, dd, *J*_1_ = 9.0, *J*_2_ = 2.4 Hz, H-7), 7.66 (1H, d, *J* = 9.0 Hz, H-8), 7.66 (2H, d, *J* = 8.4 Hz, H-2′,6′), 8.83 (2H, d, *J* = 8.4 Hz, H-3′,5′). ^13^C NMR (DMSO-*d_6_*, 75 MHz) δ_C_ 154.7 (C-2), 141.9 (C-3), 179.6 (C-4), 126.7 (C-5), 123.8 (C-6), 131.4 (C-7), 120.9 (C-8), 152.4 (C-9), 122.1 (C-10), 139.3 (C-1′), 124.6 (C-2′,6′), 124.7 (q, ^3^*J*_CF_ = 3.8 Hz, C-3′,5′), 125.1 (q, ^2^*J*_CF_ = 31.2 Hz, C-4′), 124.9 (q, ^1^*J*_CF_ = 270.7 Hz, C-7′). DCI-HRMS [M + H]^+^ calcd. for (C_16_H_9_ClF_3_O_3_)^+^: 341.0192, found: 341.0187.

#### 3.2.2. General Procedure for the Synthesis of the Dipolarophile **2**

To a solution of 3 g of the flavonol (**1**) (8.82 mmol) in anhydrous DMF, 2 equivalents of K_2_CO_3_ were added. The mixture was kept under stirring in an ice bath for 15 min. Two equivalents of propargyl bromide were then added, the reaction mixture was allowed to stir at room temperature for 2 h, and the reaction was monitored via TLC. Once the reaction was completed, the residue was diluted with water (200 mL), and the formed precipitate was then filtered through sintered glass. The dipolarophile (**2**) was obtained with an excellent yield (94%).

##### 6-Chloro-3-(prop-2-yn-1-yloxy)-2-(4-(trifluoromethyl)phenyl)-4*H*-chromen-4-one (**2**)

Yellowish solid, yield: 94%, mp: 122 °C, ^1^H NMR (CDCl_3_, 300 MHz) δ_H_ 8.24 (1H, d, *J* = 2.6 Hz, H-5), 7.67 (1H, dd, *J*_1_ = 8.9, *J*_2_ = 2.6 Hz, H-7), 7.54 (1H, d, *J* = 8.9 Hz, H-8), 7.79 (2H, d, *J* = 8.4 Hz, H-2′,6′), 8.27 (2H, d, *J* = 8.4 Hz, H-3′,5′), 5.06 (2H, d, *J* = 2.4 *Hz*, H-1″), 2.36 (1H, t, *J* = 2.4 Hz, H-3″). ^13^C NMR (CDCl_3_, 75 MHz) δ_C_ 155.4 (C-2), 139.4 (C-3), 173.9 (C-4), 125.4 (C-5), 131.4 (C-6), 134.4 (C-7), 120.0 (C-8), 153.8 (C-9), 125.2 (C-10), 134.3 (C-1′), 129.5 (C-2′,6′), 125.5 (q, ^3^*J*_CF_ = 3.8 Hz, C-3′,5′), 132.7 (q, ^2^*J*_CF_ = 32.8 Hz, C-4′), 123.9 (q, ^1^*J*_CF_ = 270.8 Hz, C-7′), 59.6 (C-1″), 78.4 (C-2″), 76.9 (C-3″).

#### 3.2.3. General Procedure for the Synthesis of 3,5-Disubstituted Trifluoromethylated Flavonoid-Based Isoxazoles **3a**–**m**

First, 50 mg of the dipolarophile (**2**), 2 equivalents of the appropriate hydroximyl chloride, 0.1 equivalent of copper iodide (CuI), and triethylamine (2 equivalents) were dissolved in 3 mL of DMF at room temperature. The mixture was subjected to microwave irradiation at 250 W for 5 min, followed by dilution with water and extraction with ethyl acetate (3 × 30 mL). The solvent was evaporated *in vacuo*, and the resulting residue was purified by silica gel column chromatography, eluted with a mixture of petroleum ether and ethyl acetate (8:2), to give the desired products (**3a**–**m**), with yields ranging from 73 to 96%.

##### 6-Chloro-3-((3-phenylisoxazol-5-yl)methoxy)-2-(4-(trifluoromethyl)phenyl)-4*H*-chromen-4-one (**3a**)

Yellowish solid, yield: 91%, mp: 134 °C, ^1^H NMR (CDCl_3_, 300 MHz) δ_H_ 8.26 (1H, d, *J* = 2.5 Hz, H-5), 7.67 (1H, m, H-7), 7.52 (1H, d, *J* = 9.0 Hz, H-8), 7.72 (2H, d, *J* = 8.4 Hz, H-2′,6′), 8.10 (2H, d, *J* = 8.4 Hz, H-3′,5′), 6.44 (1H, s, H-4″), 5.42 (2H, s, H-6″), 7.67 (2H, m, H-2‴,6‴), 7.44 (3H, m, H-3‴,4‴,5‴). ^13^C NMR (CDCl_3_, 75 MHz) δ_C_ 155.4 (C-2), 139.7 (C-3), 173.8 (C-4), 125.4 (C-5), 131.5 (C-6), 134.5 (C-7), 120.1 (C-8), 153.8 (C-9), 125.2 (C-10), 133.8 (C-1′), 129.4 (C-2′,6′), 125.6 (q, ^3^*J*_CF_ = 3.7 Hz, C-3′,5′), 132.8 (q, ^2^*J*_CF_ = 32.9 Hz, C-4′), 123.8 (q, ^1^*J*_CF_ = 270.9 Hz, C-7′), 167.8 (C-3″), 103.0 (C-4″), 162.5 (C-5″), 63.8 (C-6″), 128.7 (C-1‴), 126.9 (C-2‴,6‴), 129.1 (C-3‴,5‴), 130.3 (C-4‴).

##### 6-Chloro-3-((3-(4-fluorophenyl)isoxazol-5-yl)methoxy)-2-(4-(trifluoromethyl)phenyl)-4*H*-chromen-4-one (**3b**)

Yellowish solid, yield: 96%, mp: 147 °C, ^1^H NMR (CDCl_3_, 300 MHz) δ_H_ 8.26 (1H, d, *J* = 2.5 Hz, H-5), 7.68 (1H, dd, *J*_1_ = 8.9, *J*_2_ = 2.5 Hz, H-7), 7.52 (1H, d, *J* = 8.9 Hz, H-8), 7.73 (2H, d, *J* = 8.4 Hz, H-2′,6′), 8.11 (2H, d, *J* = 8.4 Hz, H-3′,5′), 6.41 (1H, s, H-4″), 5.41 (2H, s, H-6″), 7.66 (2H, dd, *J* = 8.6, 5.4Hz, H-2‴,6‴), 7.13 (2H, t, *J* = 8.6 Hz, H-3‴,5‴). ^13^C NMR (CDCl_3_, 75 MHz) δ_C_ 155.4 (C-2), 139.7 (C-3), 173.8 (C-4), 125.4 (C-5), 131.6 (C-6), 134.6 (C-7), 120.1 (C-8), 153.9 (C-9), 125.2 (C-10), 133.8 (C-1′), 129.4 (C-2′,6′), 125.6 (q, ^3^*J*_CF_ = 3.7 Hz, C-3′,5′), 132.8 (q, ^2^*J*_CF_ = 32.7 Hz, C-4′), 123.8 (q, ^1^*J*_CF_ = 271.1 Hz, C-7′), 168.0 (C-3″), 102.8 (C-4″), 161.6 (C-5″), 63.8 (C-6″), 124.9 (d, ^4^*J*_CF_ = 3.3 Hz, C-1‴), 128.8 (d, ^3^*J*_CF_ = 8.4 Hz, C-2‴,6‴), 116.3 (d, ^2^*J*_CF_ = 21.8 Hz, C-3‴,5‴), 164.1 (d, ^1^*J*_CF_ = 248.8 Hz, C-4‴). ESI-HRMS [M+H]^+^ calcd. for (C_26_H_15_ClF_4_NO_4_)^+^: 516.0626, found: 516.0628.

##### 6-Chloro-3-((3-(4-chlorophenyl)isoxazol-5-yl)methoxy)-2-(4-(trifluoromethyl)phenyl)-4*H*-chromen-4-one (**3c**)

Yellowish solid, yield: 94%, mp: 177 °C, ^1^H NMR (CDCl_3_, 300 MHz) δ_H_ 8.26 (1H, d, *J* = 2.5 Hz, H-5), 7.69 (1H, dd, *J*_1_ = 9.0, *J*_2_ = 2.5 Hz, H-7), 7.53 (1H, d, *J* = 9.0 Hz, H-8), 7.73 (2H, d, *J* = 8.4 Hz, H-2′,6′), 8.10 (2H, d, *J* = 8.4 Hz, H-3′,5′), 6.42 (1H, s, H-4″), 5.40 (2H, s, H-6″), 7.60 (2H, d, *J* = 8.4 Hz, H-2‴,6‴), 7.41 (2H, d, *J* = 8.4 Hz, H-3‴,5‴). ^13^C NMR (CDCl_3_, 75 MHz) δ_C_ 155.5 (C-2), 139.6 (C-3), 173.8 (C-4), 125.4 (C-5), 131.5 (C-6), 134.6 (C-7), 120.1 (C-8), 153.8 (C-9), 125.1 (C-10), 133.7 (C-1′), 129.4 (C-2′,6′), 125.6 (q, ^3^*J*_CF_ = 3.7 Hz, C-3′,5′), 132.8 (q, ^2^*J*_CF_ = 32.6 Hz, C-4′), 123.8 (q, ^1^*J*_CF_ = 272.2 Hz, C-7′), 168.1 (C-3″), 102.8 (C-4″), 161.5 (C-5″), 63.7 (C-6″), 127.1 (C-1‴), 129.4 (C-2‴,6‴), 128.1 (C-3‴,5‴), 136.4 (C-4‴). ESI-HRMS [M + H]^+^ calcd. for (C_26_H_15_Cl_2_F_3_NO_4_)^+^: 532.0330, found: 532.0332.

##### 3-((3-(4-Bromophenyl)isoxazol-5-yl)methoxy)-6-chloro-2-(4-(trifluoromethyl)phenyl)-4*H*-chromen-4-one (**3d**)

Yellowish solid, yield: 92%, mp: 179 °C, ^1^H NMR (CDCl_3_, 300 MHz) δ_H_ 8.26 (1H, d, *J* = 2.5 Hz, H-5), 7.69 (1H, dd, *J*_1_ = 9.0, *J*_2_ = 2.5 Hz, H-7), 7.53 (1H, d, *J* = 9.0 Hz, H-8), 7.73 (2H, d, *J* = 8.4 Hz, H-2′,6′), 8.11 (2H, d, *J* = 8.4 Hz, H-3′,5′), 6.43 (1H, s, H-4″), 5.41 (2H, s, H-6″), 7.58 (2H, d, *J* = 8.7 Hz, H-2‴,6‴), 7.54 (2H, d, *J* = 8.7 Hz, H-3‴,5‴). ^13^C NMR (CDCl_3_, 75 MHz) δ_C_ 155.4 (C-2), 139.7 (C-3), 173.8 (C-4), 125.4 (C-5), 131.6 (C-6), 134.6 (C-7), 120.1 (C-8), 153.9 (C-9), 125.2 (C-10), 133.8 (C-1′), 129.4 (C-2′,6′), 125.6 (q, ^3^*J*_CF_ = 3.8 Hz, C-3′,5′), 132.8 (q, ^2^*J*_CF_ = 32.8 Hz, C-4′), 123.8 (q, ^1^*J*_CF_ = 270.9 Hz, C-7′), 168.2 (C-3″), 102.7 (C-4″), 161.7 (C-5″), 63.8 (C-6″), 127.7 (C-1‴), 128.4 (C-2‴,6‴), 132.4 (C-3‴,5‴), 124.7 (C-4‴).

##### 6-Chloro-3-((3-(p-tolyl)isoxazol-5-yl)methoxy)-2-(4-(trifluoromethyl)phenyl)-4*H*-chromen-4-one (**3e**)

Yellowish solid, yield: 92%, mp: 171 °C, ^1^H NMR (CDCl_3_, 300 MHz) δ_H_ 8.27 (1H, d, *J* = 2.5 Hz, H-5), 7.68 (1H, dd, *J*_1_ = 9.0, *J*_2_ = 2.5 Hz, H-7), 7.52 (1H, d, *J* = 9.0 Hz, H-8), 7.73 (2H, d, *J* = 8.3 Hz, H-2′,6′), 8.10 (2H, d, *J* = 8.3 Hz, H-3′,5′), 6.41 (1H, s, H-4″), 5.42 (2H, s, H-6″), 7.56 (2H, d, *J* = 8.0 Hz, H-2‴,6‴), 7.24 (2H, d, *J* = 8.0 Hz, H-3‴,5‴), 2.40 (3H, s, H-7‴). ^13^C NMR (CDCl_3_, 75 MHz) δ_C_ 155.5 (C-2), 139.7 (C-3), 173.8 (C-4), 125.4 (C-5), 131.5 (C-6), 134.5 (C-7), 120.1 (C-8), 153.9 (C-9), 125.2 (C-10), 133.8 (C-1′), 129.4 (C-2′,6′), 125.6 (q, ^3^*J*_CF_ = 3.8 Hz, C-3′,5′), 132.8 (q, ^2^*J*_CF_ = 32.7 Hz, C-4′), 123.8 (q, ^1^*J*_CF_ = 271.0 Hz, C-7′), 167.6 (C-3″), 102.9 (C-4″), 162.5 (C-5″), 63.8 (C-6″), 125.9 (C-1‴), 126.8 (C-2‴,6‴), 129.8 (C-3‴,5‴), 140.5 (C-4‴), 21.6 (C-7‴).

##### 3-((3-(4-(Tert-butyl)phenyl)isoxazol-5-yl)methoxy)-6-chloro-2-(4-(trifluoromethyl)phenyl)-4*H*-chromen-4-one (**3f**)

Yellowish solid, yield: 91%, mp: 112 °C, ^1^H NMR (CDCl_3_, 300 MHz) δ_H_ 8.27 (1H, d, *J* = 2.6 Hz, H-5), 7.68 (1H, dd, *J*_1_ = 8.9, *J*_2_ = 2.6 Hz, H-7), 7.52 (1H, d, *J* = 8.9 Hz, H-8), 7.73 (2H, d, *J* = 8.4 Hz, H-2′,6′), 8.11 (2H, d, *J* = 8.4 Hz, H-3′,5′), 6.43 (1H, s, H-4″), 5.43 (2H, s, H-6″), 7.61 (2H, d, *J* = 8.4 Hz, H-2‴,6‴), 7.46 (2H, d, *J* = 8.4 Hz, H-3‴,5‴), 1.36 (9H, s, H-8‴). ^13^C NMR (CDCl_3_, 75 MHz) δ_C_ 155.5 (C-2), 139.7 (C-3), 173.8 (C-4), 125.4 (C-5), 131.5 (C-6), 134.5 (C-7), 120.1 (C-8), 153.9 (C-9), 125.2 (C-10), 133.8 (C-1′), 129.4 (C-2′,6′), 125.6 (q, ^3^*J*_CF_ = 3.7 Hz, C-3′,5′), 132.8 (q, ^2^*J*_CF_ = 32.6 Hz, C-4′), 123.8 (q, ^1^*J*_CF_ = 271.0 Hz, C-7′), 167.5 (C-3″), 103.0 (C-4″), 162.4 (C-5″), 63.8 (C-6″), 125.8 (C-1‴), 126.1 (C-2‴,6‴), 126.7 (C-3‴,5‴), 153.7 (C-4‴), 35.0 (C-7‴), 31.4 (C-8‴).

##### 6-Chloro-3-((3-(4-nitrophenyl)isoxazol-5-yl)methoxy)-2-(4-(trifluoromethyl)phenyl)-4*H*-chromen-4-one (**3g**)

Yellowish solid, yield: 73%, mp: 175 °C, ^1^H NMR (CDCl_3_, 300 MHz) δ_H_ 8.27 (1H, d, *J* = 2.6 Hz, H-5), 7.70 (1H, dd, *J*_1_ = 8.9, *J*_2_ = 2.6Hz, H-7), 7.54 (1H, d, *J* = 8.9 Hz, H-8), 7.74 (2H, d, *J* = 8.2 Hz, H-2′,6′), 8.12 (2H, d, *J* = 8.2 Hz, H-3′,5′), 6.57 (1H, s, H-4″), 5.43 (2H, s, H-6″), 7.87 (2H, d, *J* = 8.9 Hz, H-2‴,6‴), 8.31 (2H, d, *J* = 8.9 Hz, H-3‴,5‴). ^13^C NMR (CDCl_3_, 75 MHz) δ_C_ 155.4 (C-2), 139.7 (C-3), 173.8 (C-4), 125.4 (C-5), 131.7 (C-6), 134.7 (C-7), 120.1 (C-8), 153.9 (C-9), 125.2 (C-10), 133.8 (C-1′), 129.4 (C-2′,6′), 125.6 (q, ^3^*J*_CF_ = 3.9 Hz, C-3′,5′), 132.9 (q, ^2^*J*_CF_ = 33.8 Hz, C-4′), 123.8 (q, ^1^*J*_CF_ = 270.9 Hz, C-7′), 169.0 (C-3″), 102.9 (C-4″), 160.8 (C-5″), 63.8 (C-6″), 134.8 (C-1‴), 124.4 (C-2‴,6‴), 127.8 (C-3‴,5‴), 149.0 (C-4‴).

##### 6-Chloro-3-((3-(4-methoxyphenyl)isoxazol-5-yl)methoxy)-2-(4-(trifluoromethyl)phenyl)-4*H*-chromen-4-one (**3h**)

Yellowish solid, yield: 95%, mp: 152 °C, ^1^H NMR (CDCl_3_, 300 MHz) δ_H_ 8.26 (1H, d, *J* = 2.4 Hz, H-5), 7.67 (1H, dd, *J*_1_ = 9.0, *J*_2_ = 2.4 Hz, H-7), 7.51 (1H, d, *J* = 9.0 Hz, H-8), 7.72 (2H, d, *J* = 8.4 Hz, H-2′,6′), 8.10 (2H, d, *J* = 8.4 Hz, H-3′,5′), 6.38 (1H, s, H-4″), 5.40 (2H, s, H-6″), 7.60 (2H, d, *J* = 8.6 Hz, H-2‴,6‴), 6.95 (2H, d, *J* = 8.6 Hz, H-3‴,5‴), 3.86 (3H, s, H-7‴). ^13^C NMR (CDCl_3_, 75 MHz) δ_C_ 155.4 (C-2), 139.7 (C-3), 173.8 (C-4), 125.4 (C-5), 131.5 (C-6), 134.5 (C-7), 120.1 (C-8), 153.8 (C-9), 125.2 (C-10), 133.8 (C-1′), 129.4 (C-2′,6′), 125.6 (q, ^3^*J*_CF_ = 3.7 Hz, C-3′,5′), 132.8 (q, ^2^*J*_CF_ = 32.4 Hz, C-4′), 123.8 (q, ^1^*J*_CF_ = 270.7 Hz, C-7′), 167.5 (C-3″), 102.7 (C-4″), 162.1 (C-5″), 63.8 (C-6″), 121.2 (C-1‴), 128.3 (C-2‴,6‴), 114.5 (C-3‴,5‴), 161.3 (C-4‴), 55.5 (C-7‴).

##### 3-((3-(4-Butoxyphenyl)isoxazol-5-yl)methoxy)-6-chloro-2-(4-(trifluoromethyl)phenyl)-4*H*-chromen-4-one (**3i**)

Yellowish solid, yield: 94%, mp: 130 °C, ^1^H NMR (CDCl_3_, 300 MHz) δ_H_ 8.27 (1H, d, *J* = 2.5 Hz, H-5), 7.68 (1H, dd, *J*_1_ = 8.9, *J*_2_ = 2.5 Hz, H-7), 7.52 (1H, d, *J* = 8.9 Hz, H-8), 7.73 (2H, d, *J* = 8.3 Hz, H-2′,6′), 8.10 (2H, d, *J* = 8.3 Hz, H-3′,5′), 6.37 (1H, s, H-4″), 5.41 (2H, s, H-6″), 7.59 (2H, d, *J* = 8.9 Hz, H-2‴,6‴), 6.94 (2H, d, *J* = 8.9 Hz, H-3‴,5‴), 4.02 (2H, t, *J* = 6.5 Hz, H-7‴), 1.80 (2H, m, H-8‴), 1.52 (2H, m, H-9‴), 1.00 (3H, t, *J* = 7.4 Hz, H-10‴). ^13^C NMR (CDCl_3_, 75 MHz) δ_C_ 155.4 (C-2), 139.7 (C-3), 173.8 (C-4), 125.4 (C-5), 131.5 (C-6), 134.5 (C-7), 120.1 (C-8), 153.8 (C-9), 125.2 (C-10), 133.8 (C-1′), 129.4 (C-2′,6′), 125.5 (q, ^3^*J*_CF_ = 3.7 Hz, C-3′,5′), 132.7 (q, ^2^*J*_CF_ = 32.6 Hz, C-4′), 123.7 (q, ^1^*J*_CF_ = 271.5 Hz, C-7′), 167.4 (C-3″), 102.7 (C-4″), 162.2 (C-5″), 63.8 (C-6″), 120.9 (C-1‴), 128.2 (C-2‴,6‴), 115.0 (C-3‴,5‴), 160.9 (C-4‴), 68.0 (C-7‴), 31.4 (C-8‴), 19.4 (C-9‴), 14.0 (C-10‴).

##### 3-((3-(4-Butoxy-3-chlorophenyl)isoxazol-5-yl)methoxy)-6-chloro-2-(4-(trifluoromethyl)phenyl)-4*H*-chromen-4-one (**3j**)

Yellowish solid, yield: 92%, mp: 134 °C, ^1^H NMR (CDCl_3_, 300 MHz) δ_H_ 8.26 (1H, d, *J* = 2.5 Hz, H-5), 7.68 (1H, dd, *J*_1_ = 9.0, *J*_2_ = 2.5 Hz, H-7), 7.52 (1H, d, *J* = 9.0 Hz, H-8), 7.73 (2H, d, *J* = 8.3 Hz, H-2′,6′), 8.10 (2H, d, *J* = 8.3 Hz, H-3′,5′), 6.38 (1H, s, H-4″), 5.40 (2H, s, H-6″), 7.69 (1H, d, *J* = 2.2 Hz, H-2‴), 6.94 (1H, d, *J* = 8.5 Hz, H-5‴), 7.51 (1H, dd, *J*_1_ = 8.5, *J*_2_ = 2.2 Hz, H-6‴), 4.09 (2H, t, *J* = 6.5 Hz, H-7‴), 1.86 (2H, m, H-8‴), 1.56 (2H, m, H-9‴), 1.01 (3H, t, *J* = 7.4 Hz, H-10‴). ^13^C NMR (CDCl_3_, 75 MHz) δ_C_ 155.4 (C-2), 139.6 (C-3), 173.7 (C-4), 125.3 (C-5), 131.5 (C-6), 134.5 (C-7), 120.1 (C-8), 153.8 (C-9), 125.1 (C-10), 133.7 (C-1′), 129.4 (C-2′,6′), 125.5 (q, ^3^*J*_CF_ = 3.7 Hz, C-3′,5′), 132.7 (q, ^2^*J*_CF_ = 32.7 Hz, C-4′), 123.8 (q, ^1^*J*_CF_ = 271.2 Hz, C-7′), 167.8 (C-3″), 102.6 (C-4″), 161.2 (C-5″), 63.7 (C-6″), 121.6 (C-1‴), 128.6 (C-2‴), 123.6 (C-3‴), 156.2 (C-4‴), 113.2 (C-5‴), 126.3 (C-6‴), 69.1 (C-7‴), 31.2 (C-8‴), 19.3 (C-9‴), 14.0 (C-10‴).

##### 6-Chloro-3-((3-(3-chloro-4-methoxyphenyl)isoxazol-5-yl)methoxy)-2-(4-(trifluoromethyl)phenyl)-4*H*-chromen-4-one (**3k**)

Yellowish solid, yield: 92%, mp: 141 °C, ^1^H NMR (CDCl_3_, 300 MHz) δ_H_ 8.27 (1H, d, *J* = 2.5 Hz, H-5), 7.68 (1H, dd, *J*_1_ = 9.0, *J*_2_ = 2.5 Hz, H-7), 7.53 (1H, d, *J* = 9.0 Hz, H-8), 7.73 (2H, d, *J* = 8.3 Hz, H-2′,6′), 8.11 (2H, d, *J* = 8.3 Hz, H-3′,5′), 6.39 (1H, s, H-4″), 5.40 (2H, s, H-6″), 7.71 (1H, d, *J* = 2.2 Hz, H-2‴), 6.98 (1H, d, *J* = 8.6 Hz, H-5‴), 7.54 (1H, dd, *J*_1_ = 8.6, *J*_2_ = 2.2 Hz, H-6‴), 3.96 (3H, s, H-7‴). ^13^C NMR (CDCl_3_, 75 MHz) δ_C_ 155.4 (C-2), 139.7 (C-3), 173.8 (C-4), 125.4 (C-5), 131.6 (C-6), 134.6 (C-7), 120.1 (C-8), 153.9 (C-9), 125.2 (C-10), 133.8 (C-1′), 129.4 (C-2′,6′), 125.6 (q, ^3^*J*_CF_ = 3.7 Hz, C-3′,5′), 132.8 (q, ^2^*J*_CF_ = 32.7 Hz, C-4′), 123.8 (q, ^1^*J*_CF_ = 271.4 Hz, C-7′), 167.9 (C-3″), 102.6 (C-4″), 161.2 (C-5″), 63.8 (C-6″), 122.1 (C-1‴), 128.8 (C-2‴), 123.4 (C-3‴), 156.7 (C-4‴), 112.4 (C-5‴), 126.5 (C-6‴), 56.5 (C-7‴). ESI-HRMS [M + H]^+^ calcd. for (C_27_H_17_Cl_2_F_3_NO_5_)^+^: 562.0436, found: 562.0427.

##### 3-((3-(3-Bromo-4-methoxyphenyl)isoxazol-5-yl)methoxy)-6-chloro-2-(4-(trifluoromethyl)phenyl)-4*H*-chromen-4-one (**3l**)

Yellowish solid, yield: 91%, mp: 144 °C, ^1^H NMR (CDCl_3_, 300 MHz) δ_H_ 8.27 (1H, d, *J* = 2.5 Hz, H-5), 7.68 (1H, dd, *J*_1_ = 8.9, *J*_2_ = 2.5 Hz, H-7), 7.53 (1H, d, *J* = 8.9 Hz, H-8), 7.74 (2H, d, *J* = 8.3 Hz, H-2′,6′), 8.11 (2H, d, *J* = 8.3 Hz, H-3′,5′), 6.40 (1H, s, H-4″), 5.40 (2H, s, H-6″), 7.89 (1H, d, *J* = 2.1 Hz, H-2‴), 6.94 (1H, d, *J* = 8.6 Hz, H-5‴), 7.59 (1H, dd, *J*_1_ = 8.6, *J*_2_ = 2.1 Hz, H-6‴), 3.95 (3H, s, H-7‴). ^13^C NMR (CDCl_3_, 75 MHz) δ_C_ 155.4 (C-2), 139.7 (C-3), 173.8 (C-4), 125.4 (C-5), 131.6 (C-6), 134.6 (C-7), 120.1 (C-8), 153.9 (C-9), 125.2 (C-10), 133.8 (C-1′), 129.4 (C-2′,6′), 125.6 (q, ^3^*J*_CF_ = 3.7 Hz, C-3′,5′), 132.8 (q, ^2^*J*_CF_ = 33.0 Hz, C-4′), 123.9 (q, ^1^*J*_CF_ = 272.8 Hz, C-7′), 167.9 (C-3″), 102.7 (C-4″), 161.1 (C-5″), 63.8 (C-6″), 122.6 (C-1‴), 131.9 (C-2‴), 112.5 (C-3‴), 157.6 (C-4‴), 112.2 (C-5‴), 127.3 (C-6‴), 56.6 (C-7‴). DCI-HRMS [M + H]^+^ calcd. for (C_27_H_17_BrClF_3_NO_5_)^+^: 605.9931, found: 605.9937.

##### 6-Chloro-3-((3-(3,4-dimethoxyphenyl)isoxazol-5-yl)methoxy)-2-(4-(trifluoromethyl)phenyl)-4*H*-chromen-4-one (**3m**)

Yellowish solid, yield: 93%, mp: 152 °C, ^1^H NMR (CDCl_3_, 300 MHz) δ_H_ 8.25 (1H, d, *J* = 2.5 Hz, H-5), 7.67 (1H, dd, *J*_1_ = 8.9, *J*_2_ = 2.5 Hz, H-7), 7.51 (1H, d, *J* = 8.9 Hz, H-8), 7.73 (2H, d, *J* = 8.3 Hz, H-2′,6′), 8.11 (2H, d, *J* = 8.3 Hz, H-3′,5′), 6.41 (1H, s, H-4″), 5.40 (2H, s, H-6″), 7.31 (1H, d, *J* = 1.9 Hz, H-2‴), 6.89 (1H, d, *J* = 8.3 Hz, H-5‴), 7.13 (1H, dd, *J*_1_ = 8.3, *J*_2_ = 1.9 Hz, H-6‴), 3.92 (6H, s, H-7‴,8‴). ^13^C NMR (CDCl_3_, 75 MHz) δ_C_ 155.4 (C-2), 139.7 (C-3), 173.8 (C-4), 125.3 (C-5), 131.5 (C-6), 134.5 (C-7), 120.1 (C-8), 153.8 (C-9), 125.2 (C-10), 133.8 (C-1′), 129.4 (C-2′,6′), 125.5 (q, ^3^*J*_CF_ = 3.7 Hz, C-3′,5′), 132.7 (q, ^2^*J*_CF_ = 32.7 Hz, C-4′), 123.8 (q, ^1^*J*_CF_ = 271.3 Hz, C-7′), 167.5 (C-3″), 102.8 (C-4″), 162.2 (C-5″), 63.8 (C-6″), 121.4 (C-1‴), 109.4 (C-2‴), 151.0 (C-3‴), 149.6 (C-4‴), 111.3 (C-5‴), 120.1 (C-6‴), 56.1 (C-7‴), 56.1 (C-8‴).

### 3.3. α-Amylase Inhibitory Assay

The *α*-amylase inhibition assays were carried out according to the protocol described by Chortani et al. (2021) [53], with some modifications. In short, all of the compounds were dissolved in DMSO and then diluted in sodium phosphate buffer (0.02 M, pH 6.9 with 0.006 M NaCl). The level of DMSO did not exceed 1% in the mixture. The samples were tested at different concentrations of 50, 25, 12.5, and 6.25 μM. After that, 50 μL of the sample solution was added to 50 μL of *Aspergillus oryzae α*-amylase solution (0.5 mg/mL) dissolved in the same buffer medium. After pre-incubation for 10 min at 25 °C, 50 μL of starch solution (1% in buffer solution) was added to the mixture. After 10 min of incubation at 25 °C, the product of the reaction was mixed with 100 μL of 3,5-dinitrosalicylic acid solution. At this point, the test tubes were placed for 5 min in a boiling water bath (100 °C), followed by cooling to room temperature. Next, each solution was diluted with 1 mL of distilled water. Over time, the absorbance was measured at 540 nm using a Thermo Scientific™ Multiskan™ GO Microplate Spectrophotometer using a 96-well microplate.

The *α*-amylase inhibitory activity was determined as follows:

PI (%) = 100[(Abs_Control_−Abs_Sample_)/(Abs_Control_)]

where Abs_Control_ is the absorbance of the solution without sample (buffer instead of the sample solution) and Abs_Sample_ is the absorbance of the mixture with the solution of the sample to be tested. The concentration of the compound providing 50% inhibition (IC_50_) was calculated by plotting inhibition percentages against the log of concentrations. The linear regression equation of the obtained trend line was displayed to determine the IC_50_. Acarbose was used as a positive control. Each assay was conducted in triplicate.

### 3.4. Molecular Docking Procedure

The chemical compound structures of acarbose and the synthesized compounds (**1**, **2**, and **3a**–**m**) were generated and optimized using ACD (3D viewer) software [54], where their energies were minimized. The crystal structure of *Aspergillus oryzae α*-amylase protein (PDB: 7TAA) was obtained from the RSCB databank [55]. The protein was prepared by removing the complexed inhibitor ligand and water molecules. Then, the polar hydrogens were added, followed by appending Kollman charges. Hence, the grid box with dimensions of 65 × 52 × 52 points, spacing of 0.375 Å, and centered with coordinates x: 38.725, y: 44.127, and z: 25.633 was generated based on the acarbose binding position at the target protein binding site. The molecular docking analyses of acarbose and the synthesized compounds (**1**, **2**, and **3a**–**m**) were performed using AutoDock Vina software [56]. Molecule–enzyme interactions were drawn and construed by employing the BIOVIA Discovery Studio Visualizer D.S. (2017) (BIOVIA, San Diego, CA, USA).

NMR spectra of the synthesized compounds (**1**, **2** and **3a**–**m**) are available in Supplementary Materials.

### 3.5. Statistical Analysis

Statistical analysis was performed using GraphPad Prism 7.0 (GraphPad Software Inc., San Diego, CA, USA). All of the data are presented as the mean ± standard error of the mean (SEM). The difference between two groups was evaluated using Student’s *t*-test. Significant difference among three or more groups was determined by one-way ANOVA with a post hoc analysis (Tukey’s test).

## 4. Conclusions

The 15newly synthesized trifluoromethylated flavonoid-based isoxazoles (**3a**–**m**) were evaluated in vitro for their *α*-amylase inhibitory activity, and considerable results were noted. The cycloadducts **3b**, **3h**, **3j**, and **3m** exhibited comparable efficacy and potency to those of acarbose used as standard substance to inhibit the *α*-amylase enzyme. In addition, the analysis of the structure–activity relationship revealed that halogenated and alkoxylated derivatives were the most active compounds. According to the in silico docking analysis, the trifluoromethylated flavonoid-based isoxazoles showed low binding energy, a large number of interactions, and the correct binding position in the active pocket of the target enzyme. Most of them interacted with the nucleophile and the catalytic acid/base residues (GLU-230 and ASP-206), which may explain their efficacy and potency in inhibiting the enzymatic activity of *α*-amylase. The promising activities displayed by the synthesized hybrid compounds encourage their involvement as candidates for the discovery of new antidiabetic and anti-obesity agents. However, very in-depth studies and safety factors must take into account the risks associated with the novelty of the active substance, toxicity, stability, and solubility in the aqueous medium, and pharmacodynamic characteristics, including the reliability with which side effects can be monitored in humans before potentially serious/irreversible effects develop.

## Figures and Tables

**Table 1:** Structures and yields of hydroximyl chlorides (**a**–**m**) and trifluoromethylated flavonoid-based isoxazoles (**3a**–**m**).

Entry	R_1_	R_2_	Yield (%)	Yield (%)
1	H	H	a (90)	**3a** (91)
2	F	H	b (98)	**3b** (96)
3	Cl	H	c (96)	**3c** (94)
4	Br	H	d (93)	**3d** (92)
5	CH_3_	H	e (92)	**3e** (92)
6	*t*-Bu	H	f (92)	**3f** (91)
7	NO_2_	H	g (80)	**3g** (73)
8	OMe	H	h (97)	**3h** (95)
9	O-*n*-Bu	H	i (96)	**3i** (94)
10	O-*n*-Bu	Cl	j (96)	**3j** (92)
11	OMe	Cl	k (95)	**3k** (92)
12	OMe	Br	l (95)	**3l** (91)
13	OMe	OMe	m (95)	**3m** (93)

**Table 2:** *α*-Amylase inhibition potency (IC_50_ ± SEM μM) and efficacy (PI at 50 μM), binding energy (kcal/mol), and interaction detail of compounds **1**, **2**, and **3a**–**m** docked in the active site of the *α*-amylase enzyme (PDB: 7TAA).

Compound	*α*-Amylase Inhibition		Binding Energy (kcal/mol)	Interaction Detail: NI/NIAA: IAA
	IC_50_ ± SEM μM	PI ± SEM%		
**1**	15.3 ± 0.4 ^c^	75.2 ± 1.4 ^d^	−7.7	12/8: GLN-35 *, TYR-75, HIS-80, TYR-82, HIS-122 *, ASP-206, ASP-340, ARG-344 *
**2**	17.7 ± 0.2 ^d^	75.2 ± 0.3 ^d^	−7.8	16/7: TYR-75, HIS-80, TYR-82, HIS-122*, ASP-168, ASP-206, ASP-340
**3a**	27.2 ± 0.7 ^g^	65.6 ± 0.2 ^f^	−9.6	11/7: GLN-35 *, TYR-75, HIS-80, TYR-82, LEU-166, ASP-340, ARG-344 **
**3b**	12.6 ± 0.2 ^a^	94.7 ± 1.2 ^a^	−9.6	16/10: GLN-35 *, TYR-75, HIS-80, TYR-82, LEU-166, GLY-167 *, LEU-173, ASP-206, ASP-340, ARG-344 **
**3c**	14.4 ± 0.2 ^bc^	87.1 ± 0.7 ^b^	−8.6	16/11: TYR-75, TYR-82, LEU-166, LEU-173, ASP-206, HIS-210, LEU-232,HIS-296 *, ASP-297, ASP-340, ARG-344
**3d**	14.6 ± 0.3 ^c^	85.4 ± 0.9 ^b^	−8.6	17/12: TYR-75, TYR-82, TRP-83 *, LEU-166, LEU-173, ASP-206, HIS-210, LEU-232,HIS-296 *, ASP-297, ASP-340, ARG-344
**3e**	26.0 ± 0.7 ^g^	65.4 ± 0.4 ^f^	−9.6	12/8: GLN-35 *, TYR-75, TYR-82, LEU-166, GLY-167 *, LEU-173, ASP-340, ARG-344 **
**3f**	27.6 ± 1.1 ^g^	64.5 ± 0.7 ^f^	−8.2	12/8: GLN-35 *, TYR-75, HIS-80 *, TYR-82, TRP-83, GLY-167 *, ASP-340, ARG-344
**3g**	18.1 ± 0.3 ^d^	74.7 ± 0.3 ^d^	−9.2	11/9: GLN-35 *, TYR-79 *, TYR-82, TRP-83, TYR-155, ASP-206, LEU-232, ASP-297, ASP-340
**3h**	13.3 ± 0.2 ^b^	93.5 ± 1.1 ^a^	−9.3	15/10: GLN-35 *, TYR-75, HIS-80, TYR-82, LEU-166, GLY-167 *, LEU-173, GLU-230, ASP-340, ARG-344 **
**3i**	15.6 ± 0.2 ^c^	79.4 ± 1.0 ^c^	−8.7	17/10: GLN-35 *, TYR-75, HIS-80, LEU-166, GLY-167 *, LEU-173, HIS-210, ASP-297, ASP-340, ARG-344 **
**3j**	13.7 ± 0.2 ^b^	93.1 ± 0.9 ^a^	−9.2	16/13: GLN-35 *, TYR-75, HIS-80, TYR-82, LEU-166, GLY-167 *, LEU-173, HIS-210, GLU-230, LEU-232, HIS-296, ASP-340, ARG-344 **
**3k**	21.0 ± 0.6 ^e^	72.5 ± 1.1 ^de^	−9.0	13/11: GLN-35 *, TYR-75, HIS-80, TYR-82, TRP-83 *, LEU-166, GLY-167 *, LEU-173, ASP-297, ASP-340, ARG-344
**3l**	23.0 ± 0.2 ^f^	70.4 ± 0.4 ^e^	−8.8	12/10: GLN-35 *, TYR-75, HIS-80, TYR-82, LEU-166, GLY-167 *, LEU-173, ASP-297, ASP-340, ARG-344
**3m**	13.8 ± 0.1 ^b^	93.3 ± 2.0 ^a^	−9.1	13/10: TYR-75, HIS-80 *, TYR-82, TYR-155, LEU-166, GLY-167 *, LEU-173, ASP-206, LEU-232, ASP-340
Acarbose	12.4 ± 0.1 ^a^	97.8 ± 0.5 ^a^	−7.9	11/9: GLN-35 *, HIS-80 *, TRP-83 *, ASP-206 *, LYS-209 *, GLU-230 *, LEU-232 *, ASP-297 *, ASP-340 *

NI: number of interactions; NIAA: number of interacting amino acids; IAA: interacting amino acids; * = one hydrogen bond; ** = Two hydrogen bonds. *α*-Amylase inhibitory activity as IC_50_ ± SEM μM is the compound concentration providing 50% of inhibition (potency parameter), while PI ± SEM% is the percentage inhibition at the concentration of 50 μM (Efficacy parameter). The IC_50_ and PI values cited in the same column and bearing the same letter are not significantly different at *p*-values less than 0.05.

## Data Availability

Not applicable.

## Supplementary Materials

The following are available online, NMR Spectra of the synthesized compounds (**1**, **2** and **3a**–**m**), Table S1: The concentration-response curves used to calculate the IC_50_ values.

## Sample Availability

Samples of the compounds are not available from the authors.
