# Supplementary material for: Trifluoromethylated Flavonoid-Based Isoxazoles as Antidiabetic and Anti-Obesity Agents: Synthesis, In Vitro α-Amylase Inhibitory Activity, Molecular Docking and Structure–Activity Relationship Analysis
Source: Molecules. 2021 Aug 27;26(17):5214. doi: 10.3390/molecules26175214 (PMC8434401; doi:10.3390/molecules26175214)
Supplement: Supplementary file 1 [file molecules-26-05214-s001.zip › molecules-1311610-supplementary.pdf]

molecules

Article

Trifluoromethylated Flavonoid-Based Isoxazoles as Antidiabetic and Anti-Obesity Agents: Synthesis, *In Vitro* α-Amylase Inhibitory Activity, Molecular Docking, and Structure–Activity Relationship Analysis

Faisal K. Algethami [1,*], Ilyes Saidi [2], Hani Nasser Abdelhamid [3], Mohamed R. Elamin [1], Babiker Y. Abdulkhair [1], Amani Chrouda [4] and Hichem Ben Jannet [2,*]

1 Department of Chemistry, College of Science, Imam Mohammad Ibn Saud Islamic University (IMSIU), Riyadh 11432, Saudi Arabia; mohamedrahmt99@gmail.com (M.R.E.); babiker35.by@gmail.com(B.Y.A.)

2 Laboratory of Heterocyclic Chemistry, Natural Products and Reactivity (LR11ES39), Medicinal Chemistry and Natural Products Team, Faculty of Science of Monastir, University of Monastir, Avenue of Environment, Monastir 5019, Tunisia; sisoelyesaidi@live.fr

3 Department of Chemistry, Advanced Multifunctional Materials Laboratory, Faculty of Science, Assiut University, Assiut 71575, Egypt; chemist.hani@yahoo.com

4 Department of Chemistry, College of Science Al-zulfi, Majmaah University, Al-Majmaah 11952, Saudi Arabia; amain.c@mu.edu.sa

* Correspondence: falgethami@imamu.edu.sa (F.K.A.); hichem.bjannet@gmail.com (H.B.J.)

Figure S1: NMR spectra of the synthesized compounds (1, 2 and 3a-m).

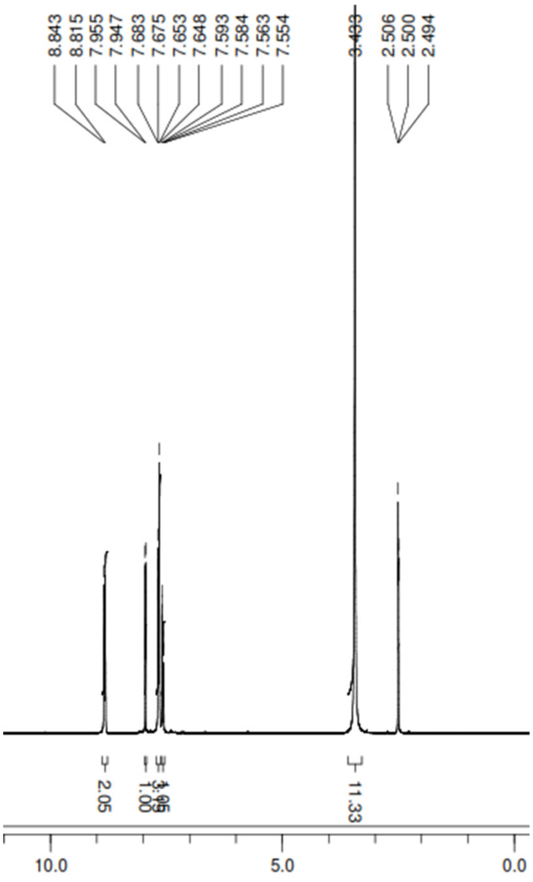

^{1}H NMR Spectrum (300 MHz, DMSO-d_6) of 1.

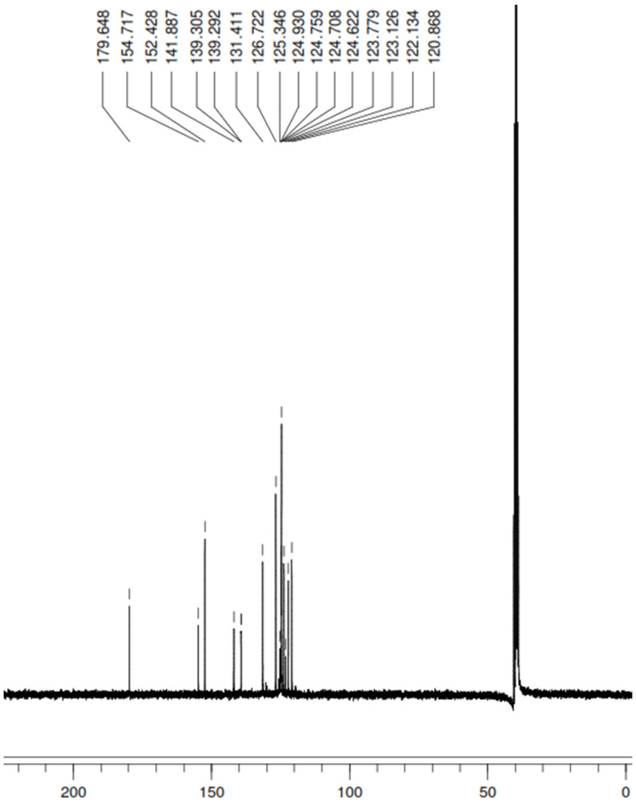

^{13}C NMR Spectrum (75 MHz, DMSO-*d_6*) of 1.

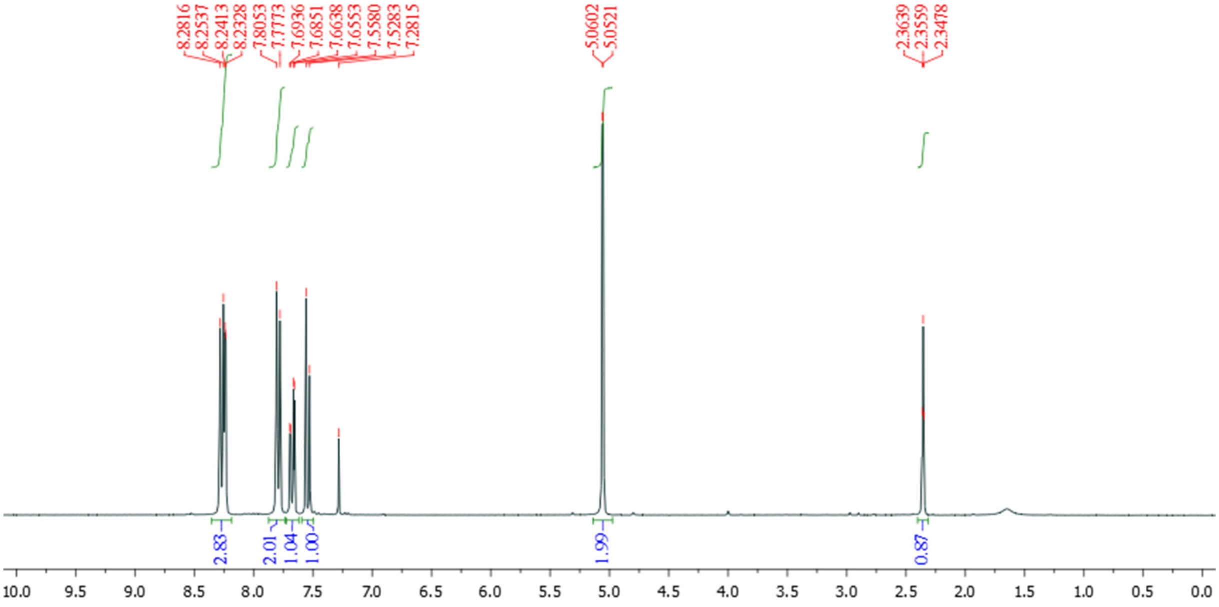

^{1}H NMR Spectrum (300 MHz, $CDCl_3$) of 2.

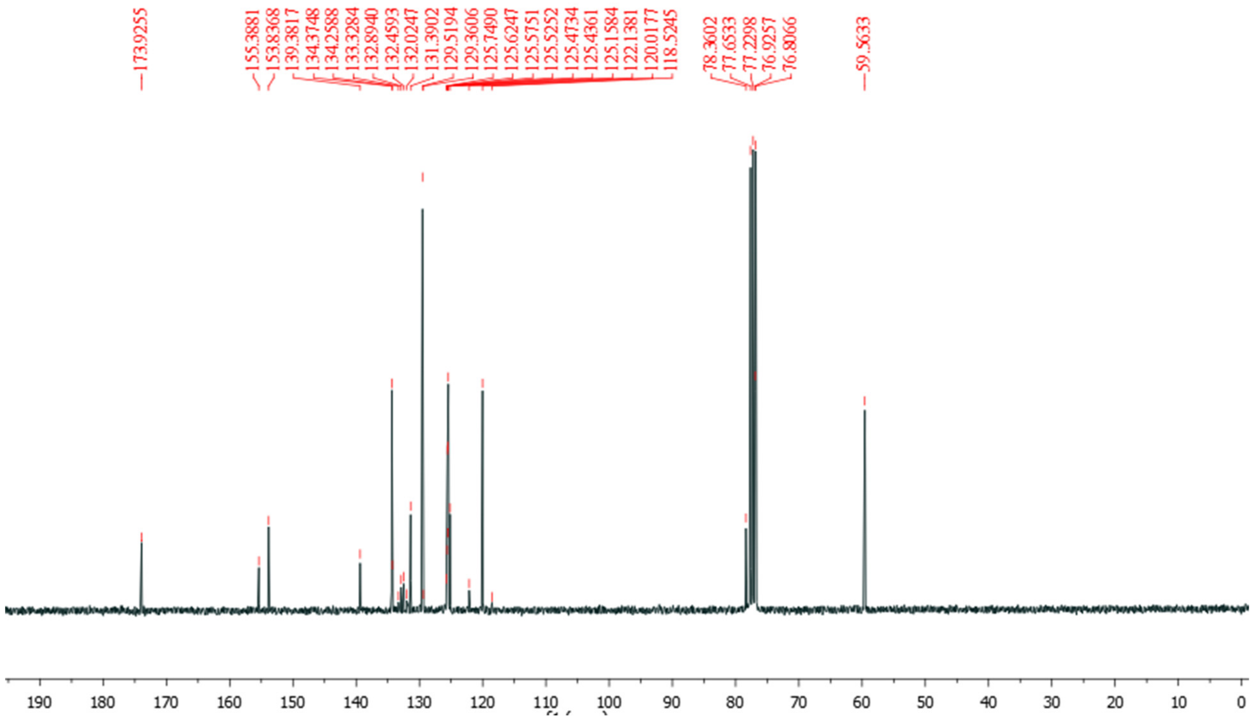

^{13}C NMR Spectrum (75 MHz, $CDCl_3$) of 2.

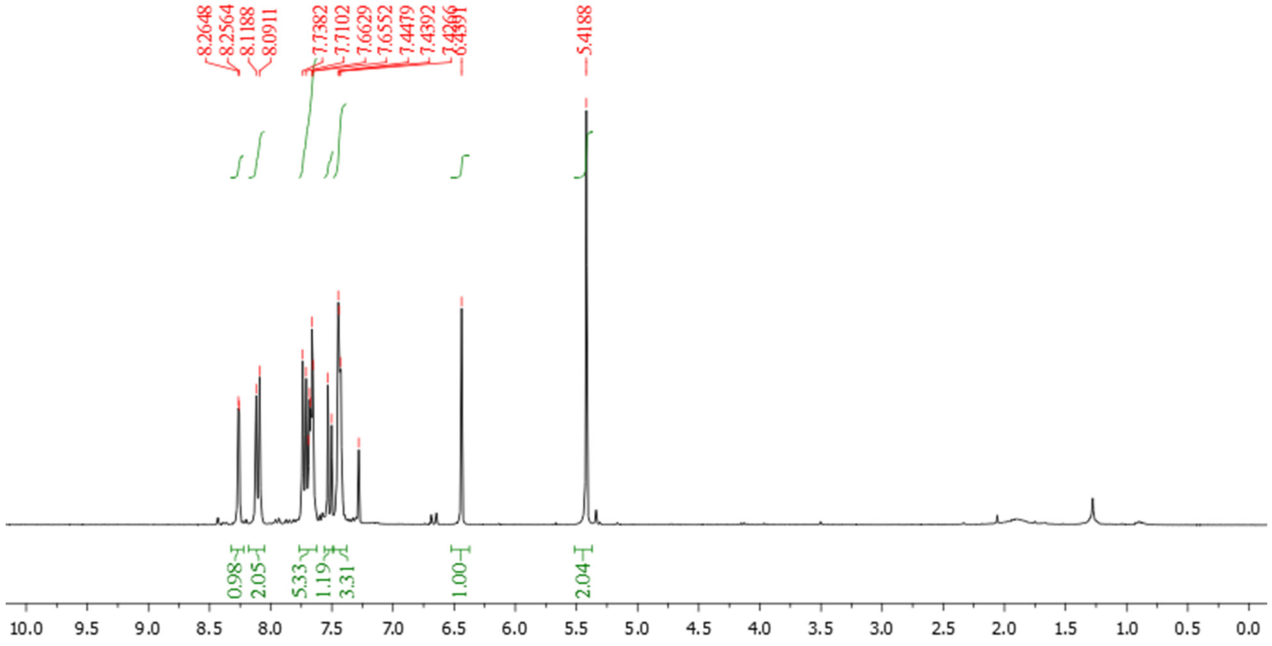

^{1}H NMR Spectrum (300 MHz, $CDCl_3$) of 3a.

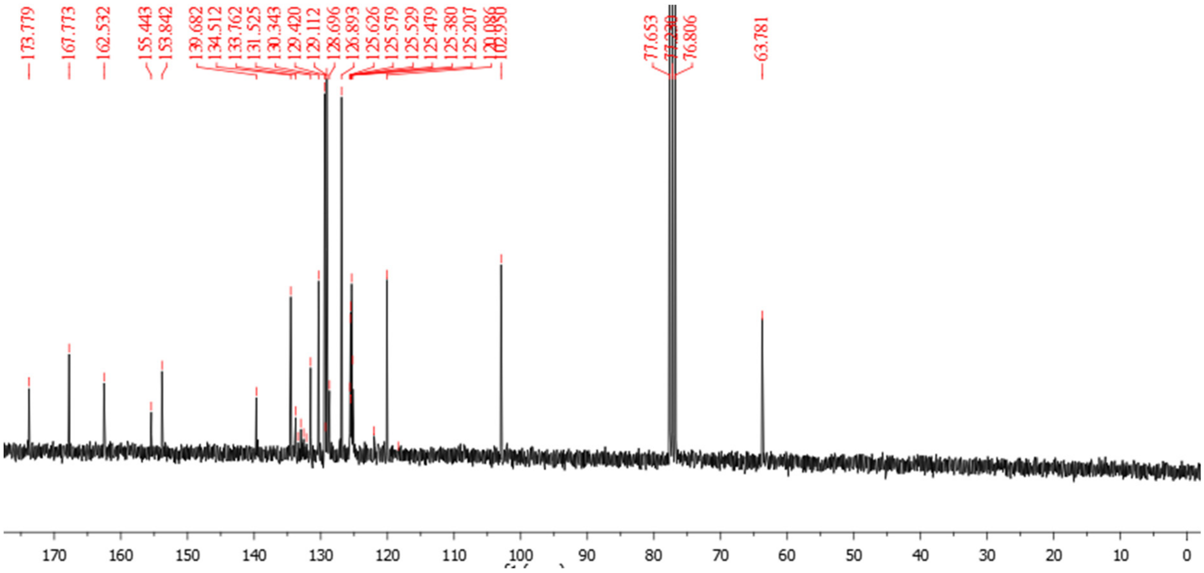

^{13}C NMR Spectrum (75 MHz, $CDCl_3$) of 3a.

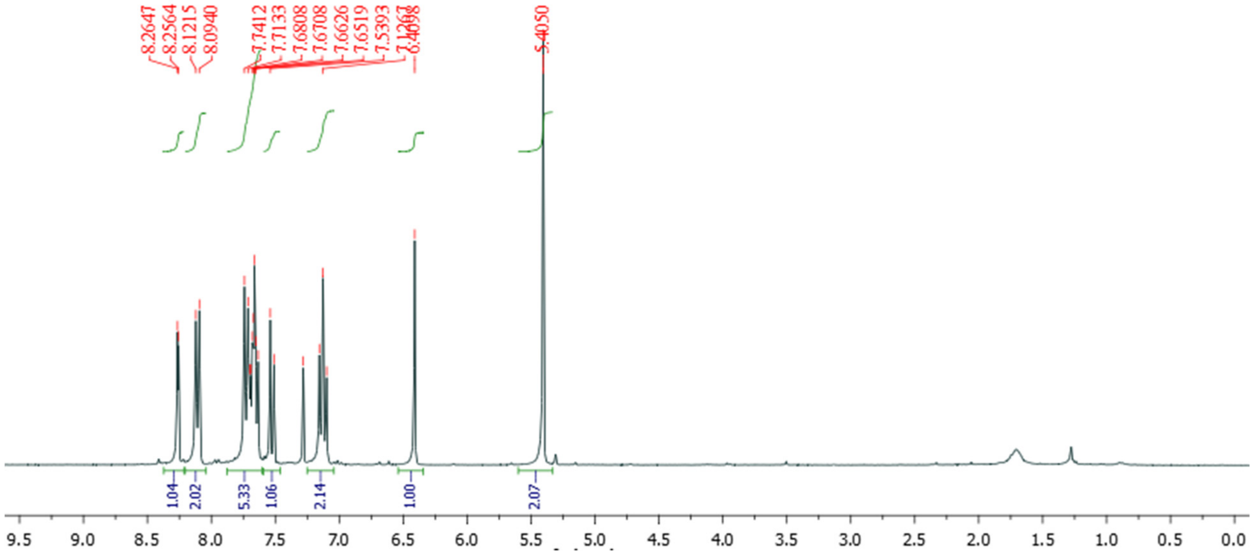

^{1}H NMR Spectrum (300 MHz, $CDCl_3$) of 3b.

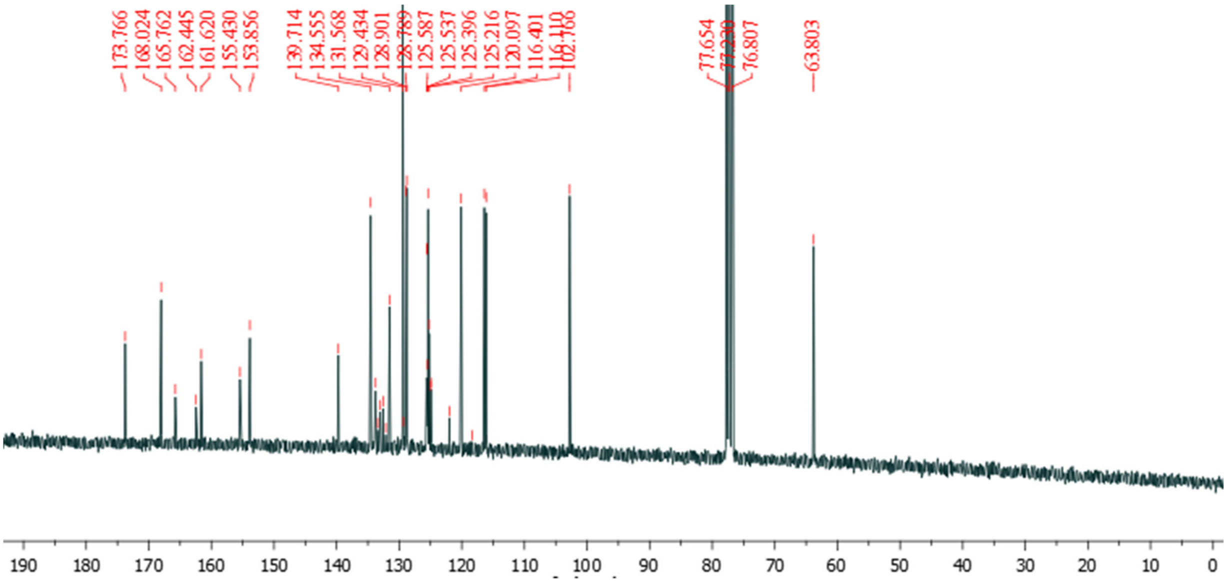

^{13}C NMR Spectrum (75 MHz, $CDCl_3$) of 3b.

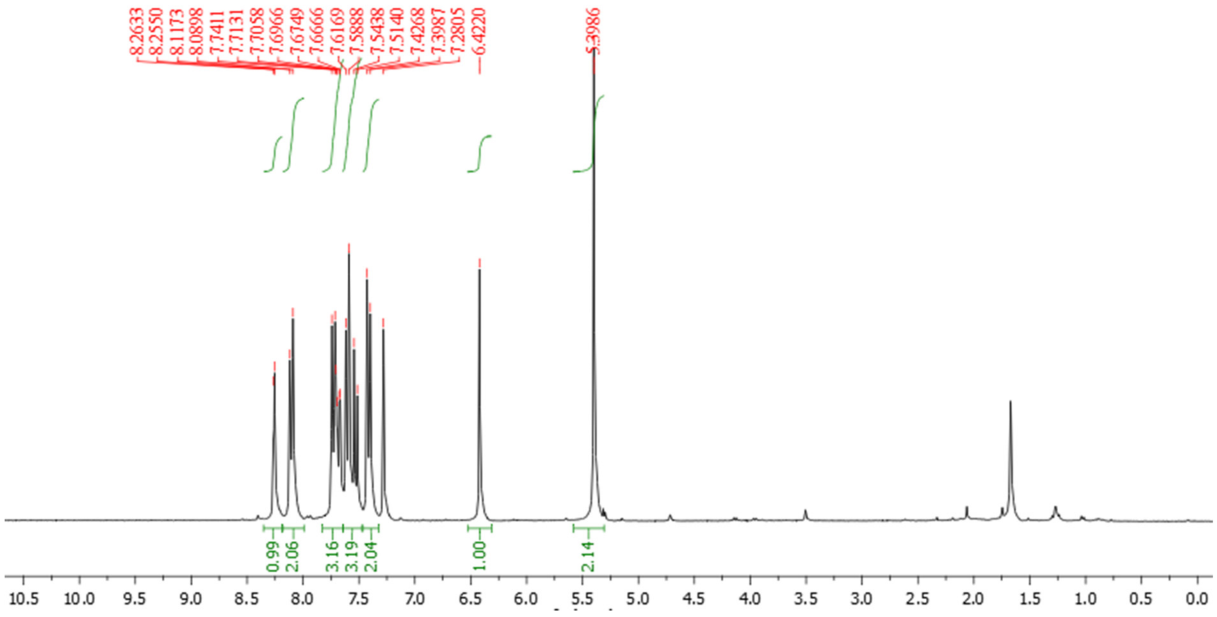

^{1}H NMR Spectrum (300 MHz, $CDCl_3$) of 3c.

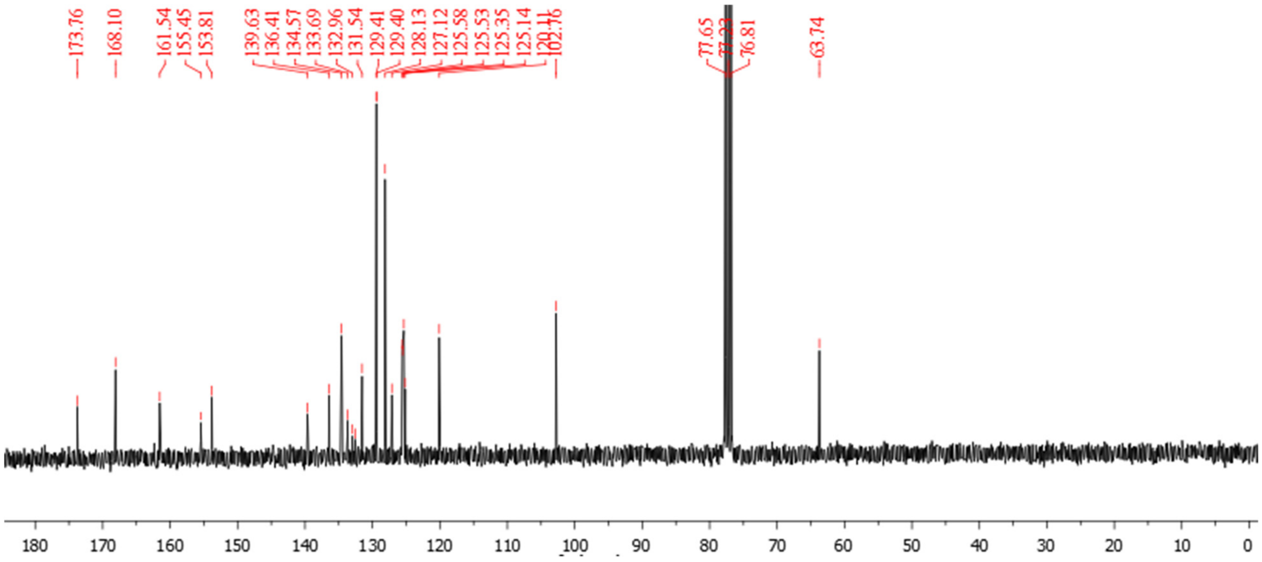

^{13}C NMR Spectrum (75 MHz, $CDCl_3$) of 3c.

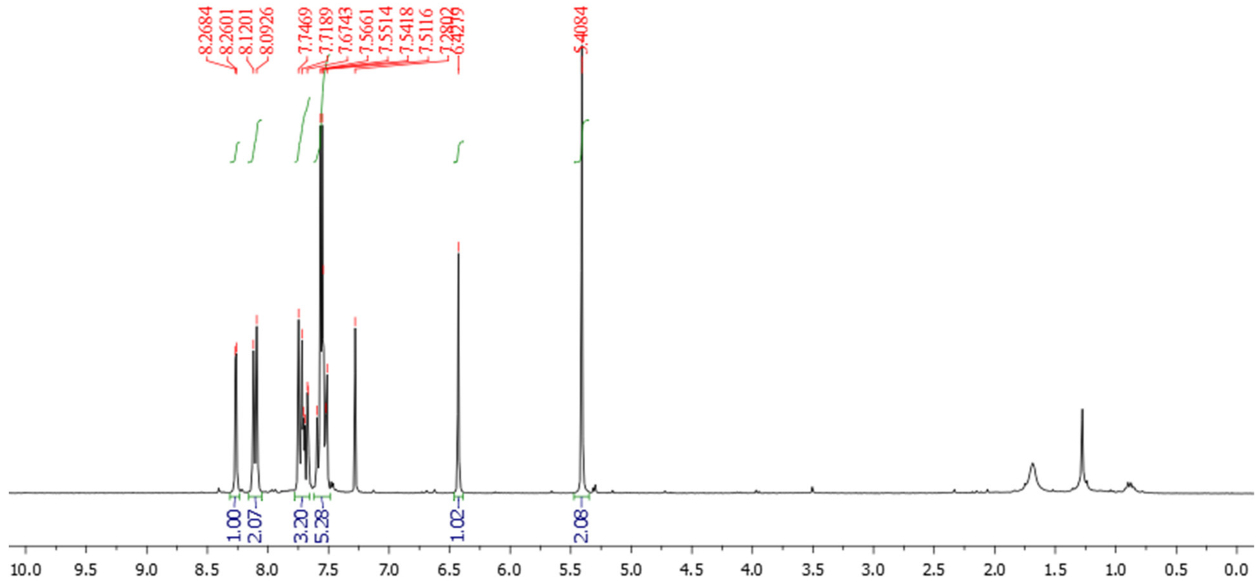

¹H NMR Spectrum (300 MHz, CDCl₃) of 3d.

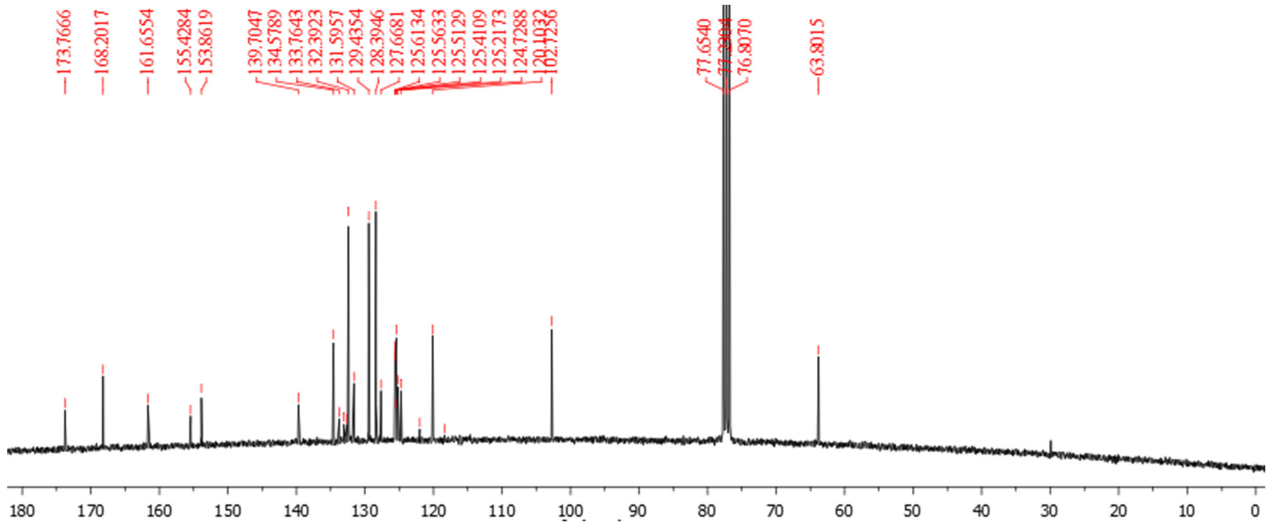

¹³C NMR Spectrum (75 MHz, CDCl₃) of 3d.

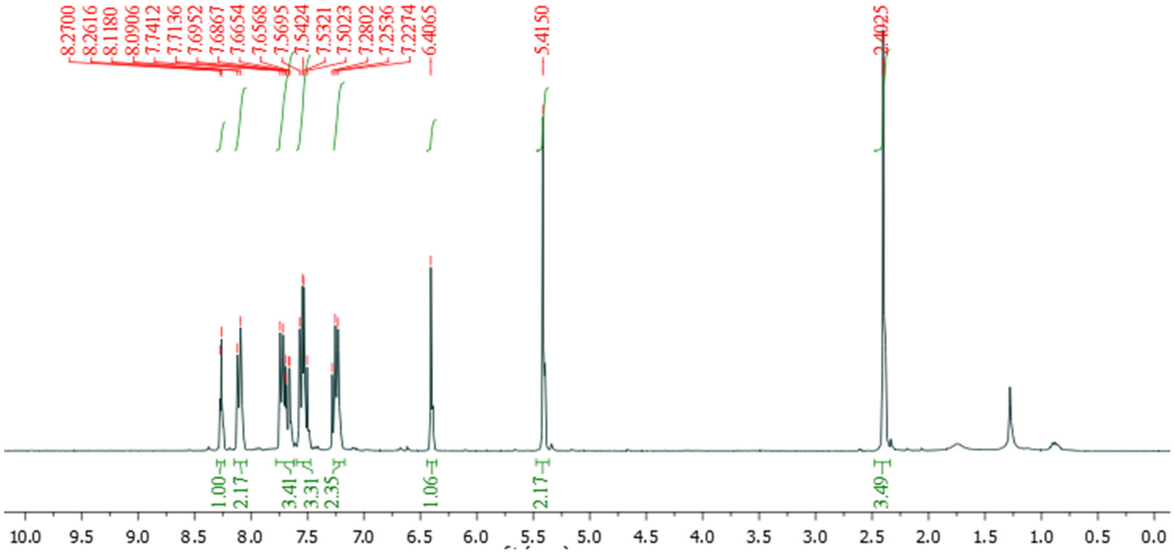

¹H NMR Spectrum (300 MHz, CDCl₃) of 3e.

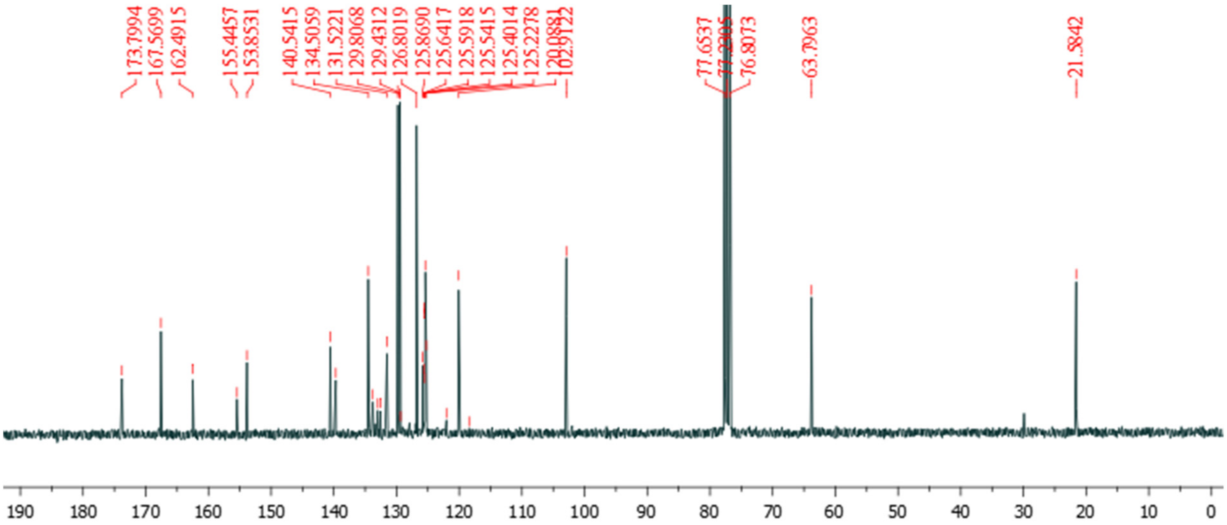

^{13}C NMR Spectrum (75 MHz, $CDCl_3$) of 3e.

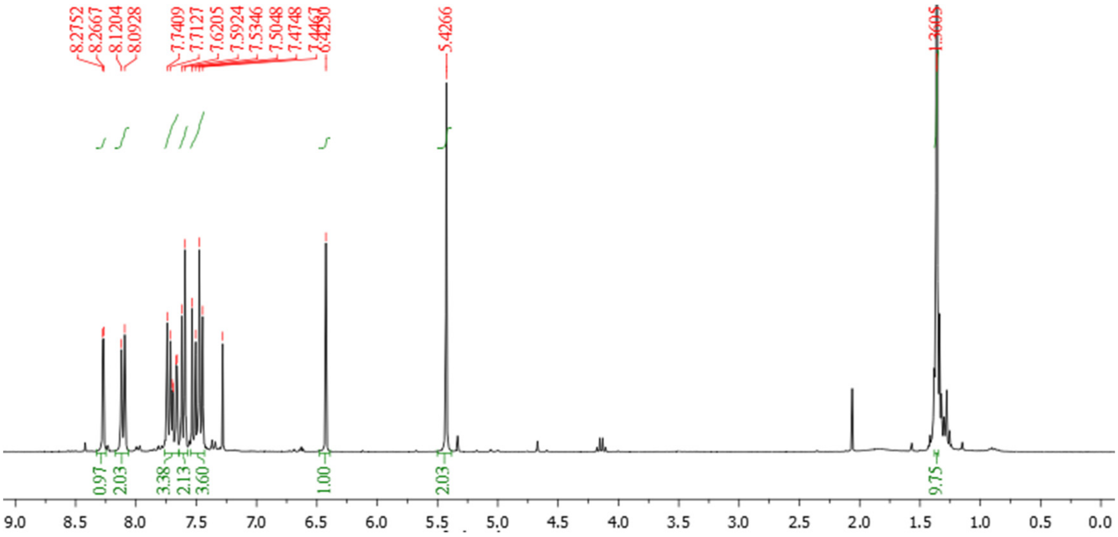

^{1}H NMR Spectrum (300 MHz, $CDCl_3$) of 3f.

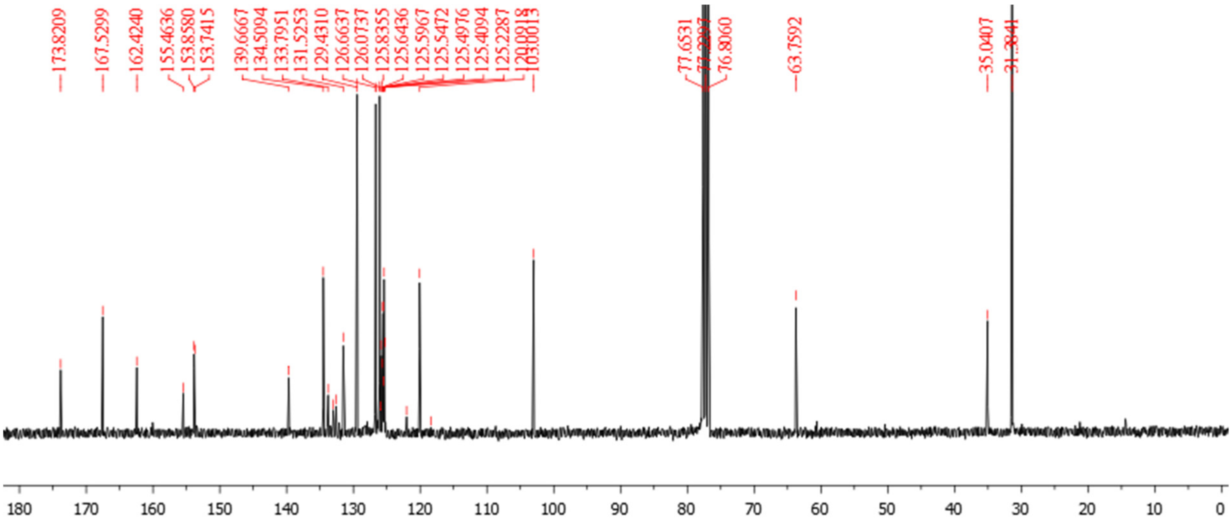

^{13}C NMR Spectrum (75 MHz, $CDCl_3$) of 3f.

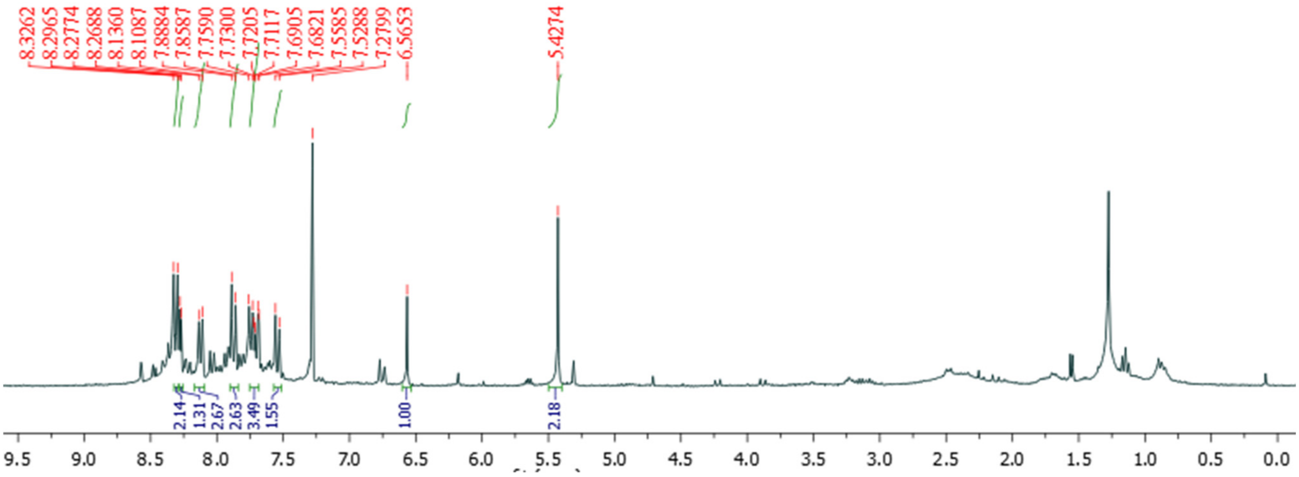

^{1}H NMR Spectrum (300 MHz, CDCl3) of 3g.

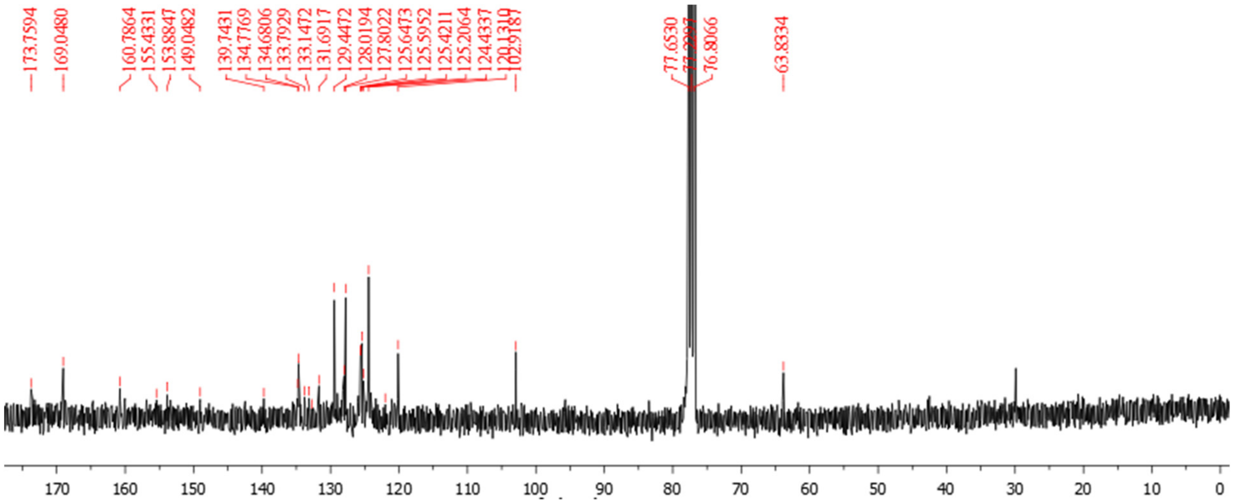

^{13}C NMR Spectrum (75 MHz, CDCl3) of 3g.

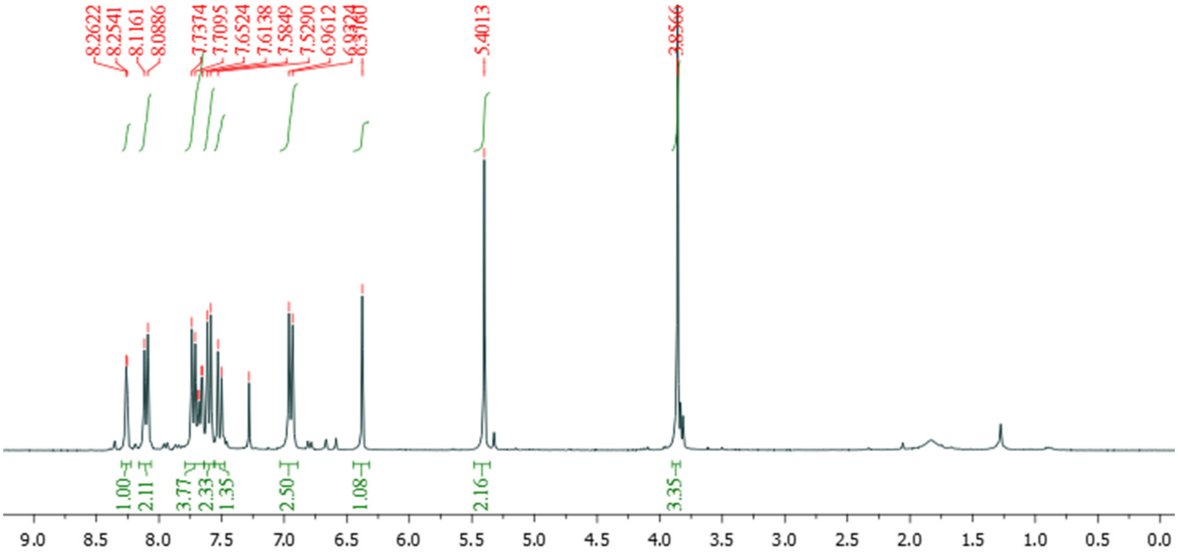

^{1}H NMR Spectrum (300 MHz, CDCl3) of 3h.

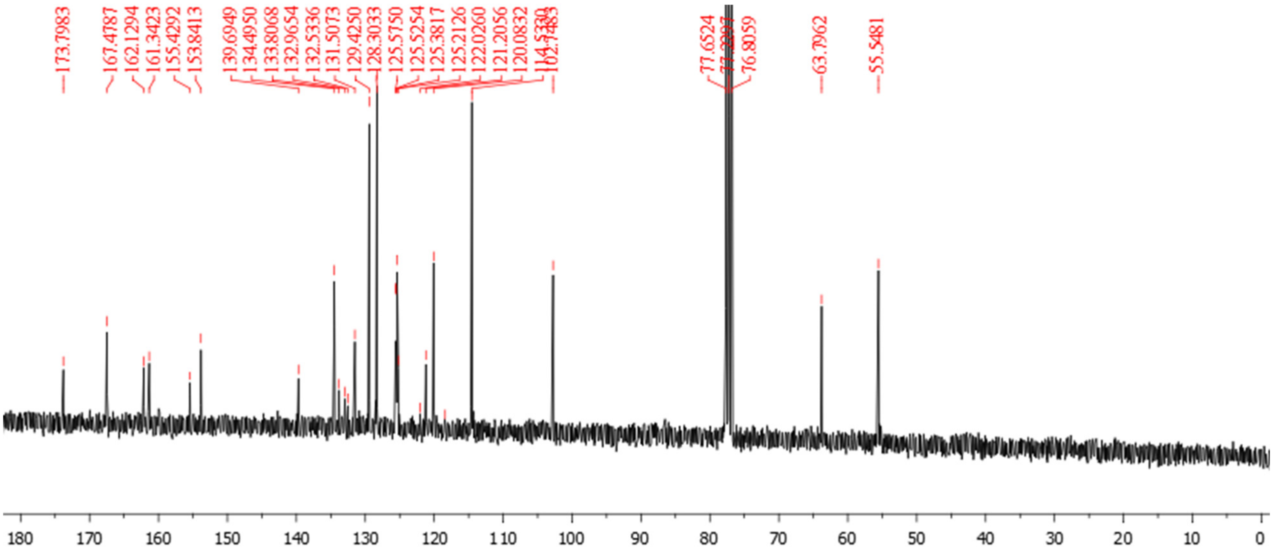

^{13}C NMR Spectrum (75 MHz, $CDCl_3$) of 3h.

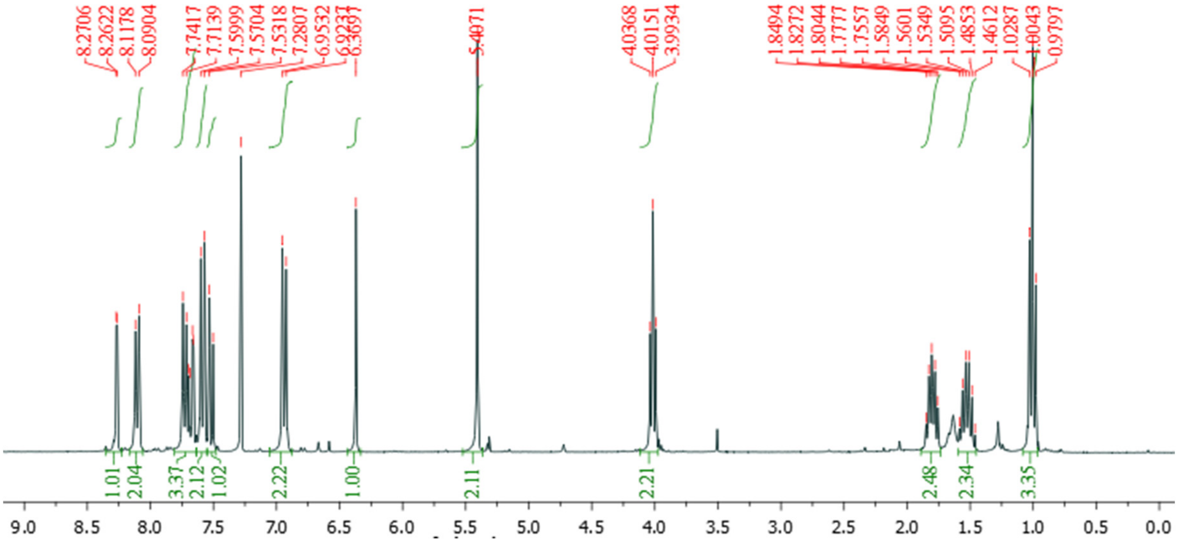

^{1}H NMR Spectrum (300 MHz, $CDCl_3$) of 3i.

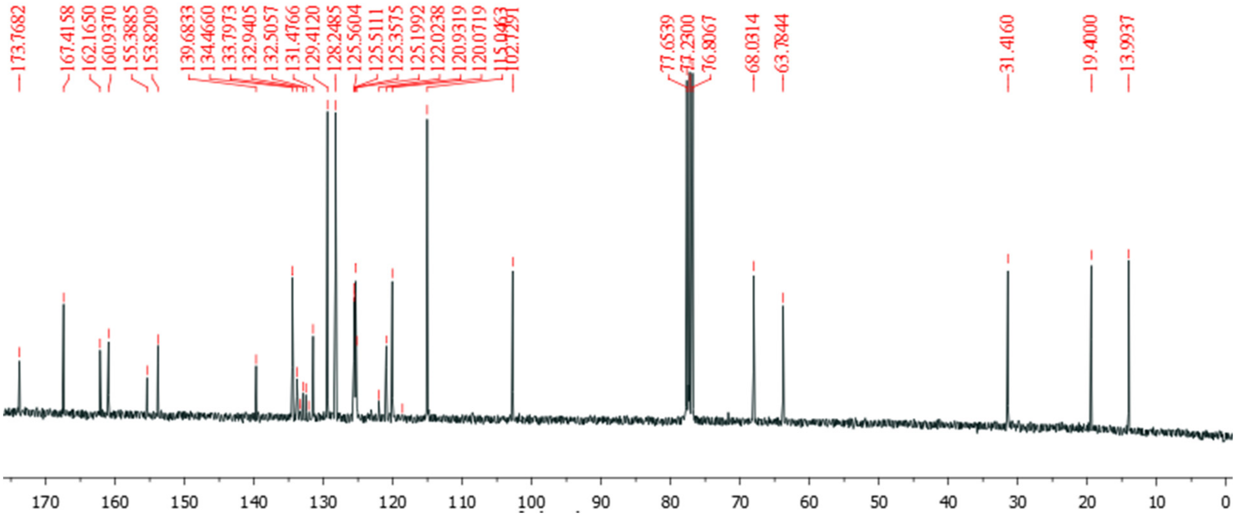

^{13}C NMR Spectrum (75 MHz, $CDCl_3$) of 3i.

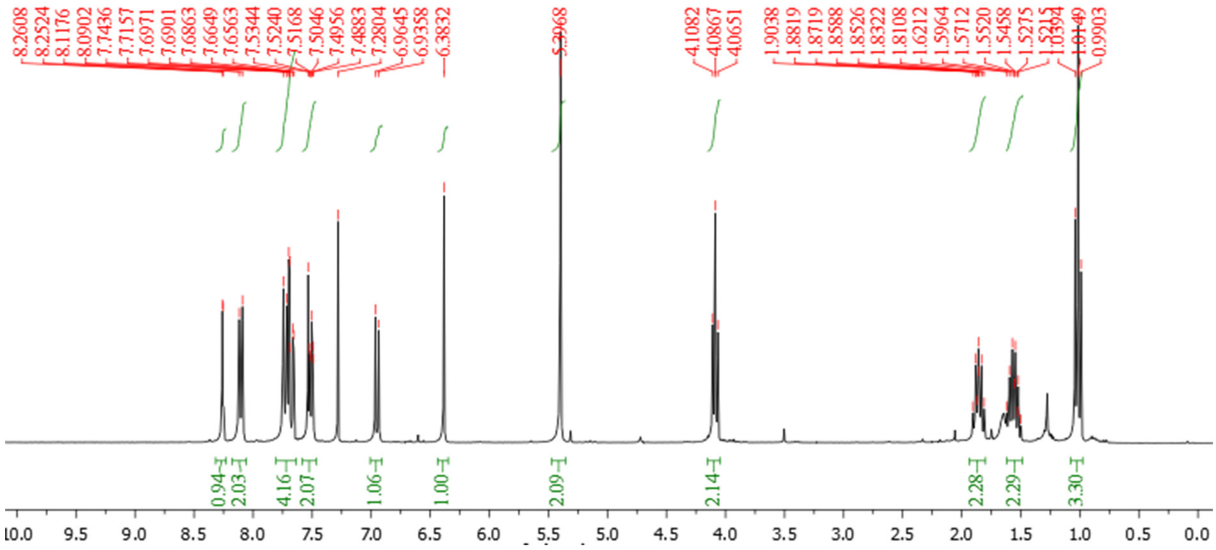

¹H NMR Spectrum (300 MHz, $CDCl_3$) of 3j.

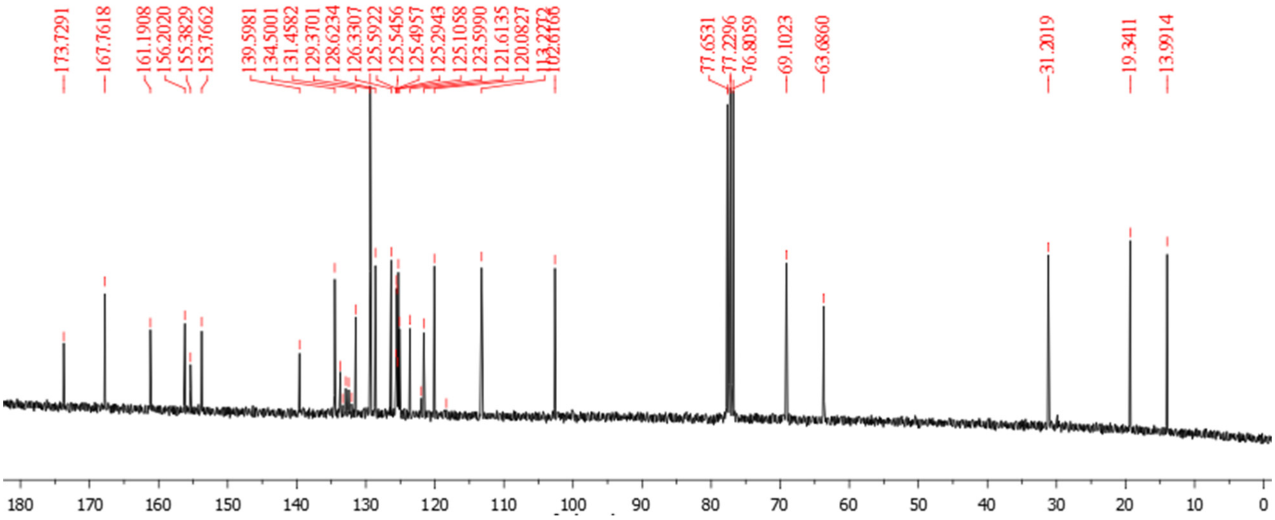

¹³C NMR Spectrum (75 MHz, $CDCl_3$) of 3j.

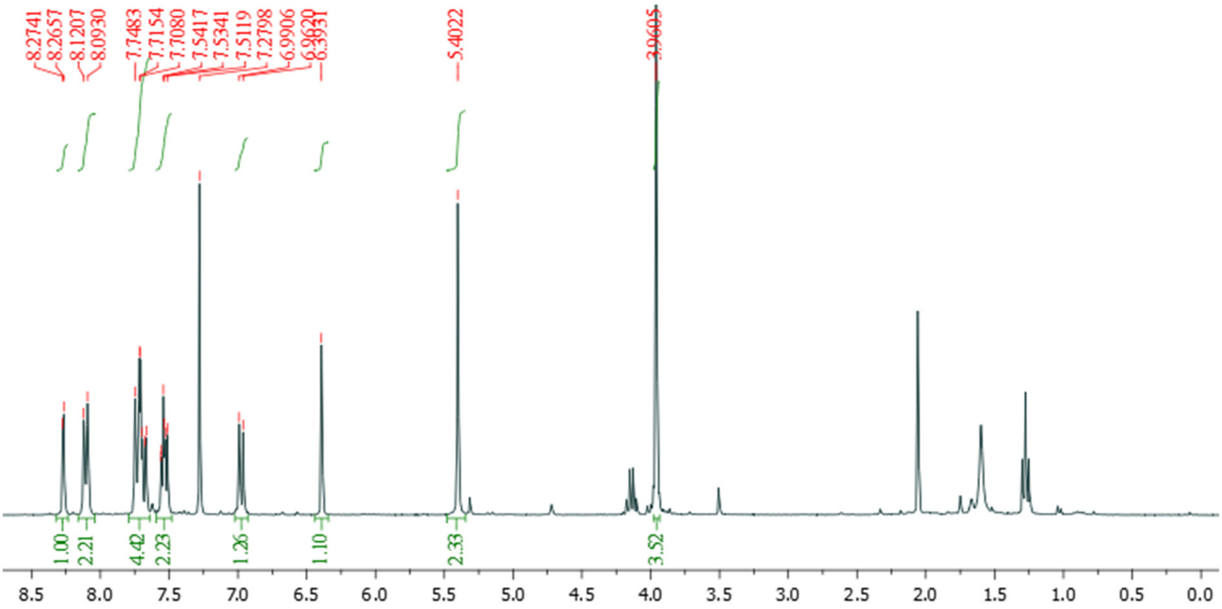

¹H NMR Spectrum (300 MHz, $CDCl_3$) of 3k.

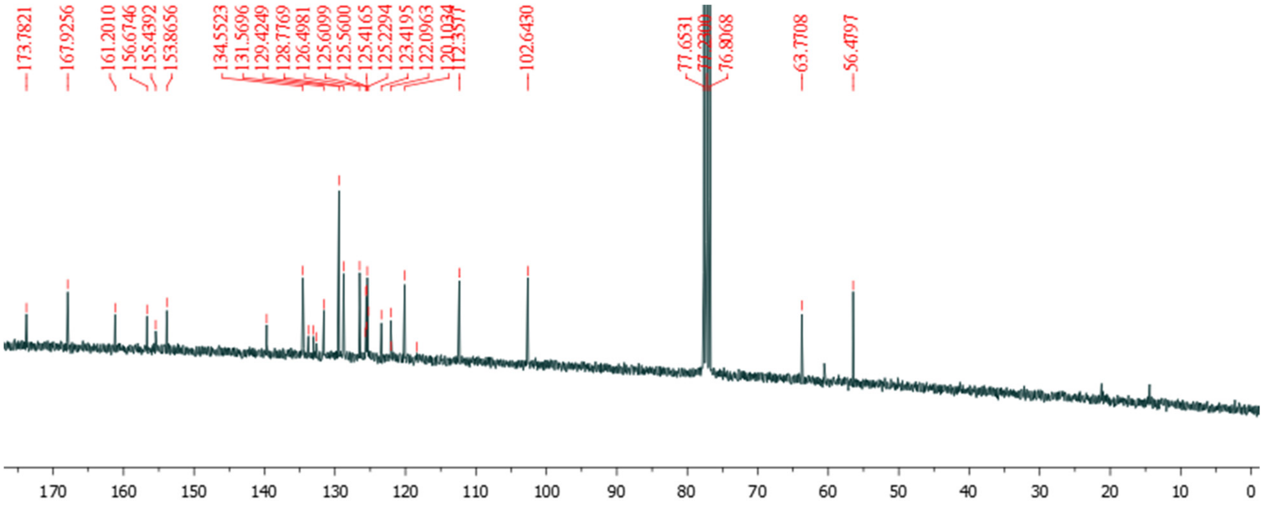

^{13}C NMR Spectrum (75 MHz, CDCl3) of 3k.

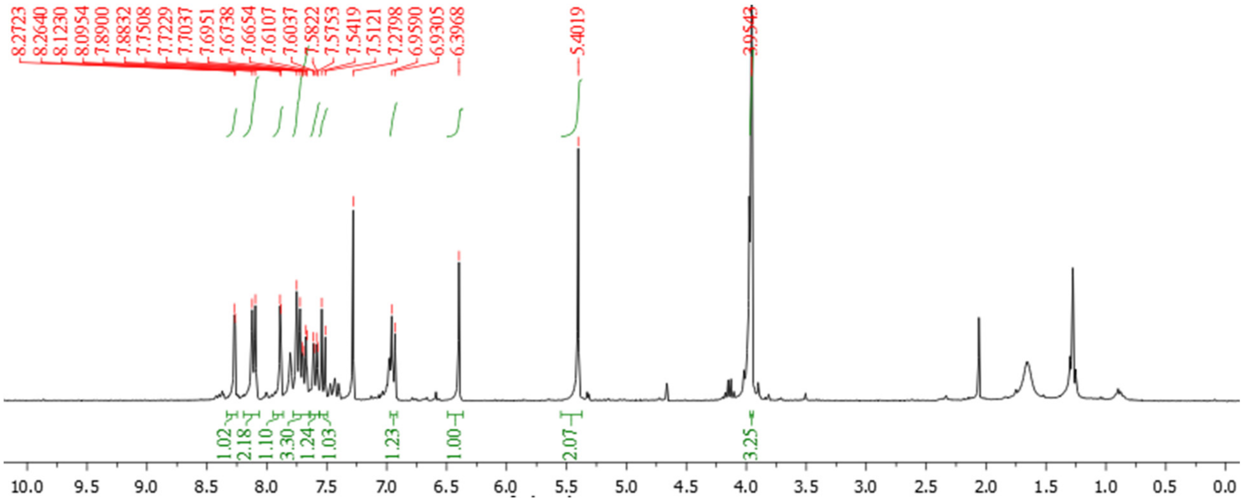

^{1}H NMR Spectrum (300 MHz, CDCl3) of 3l.

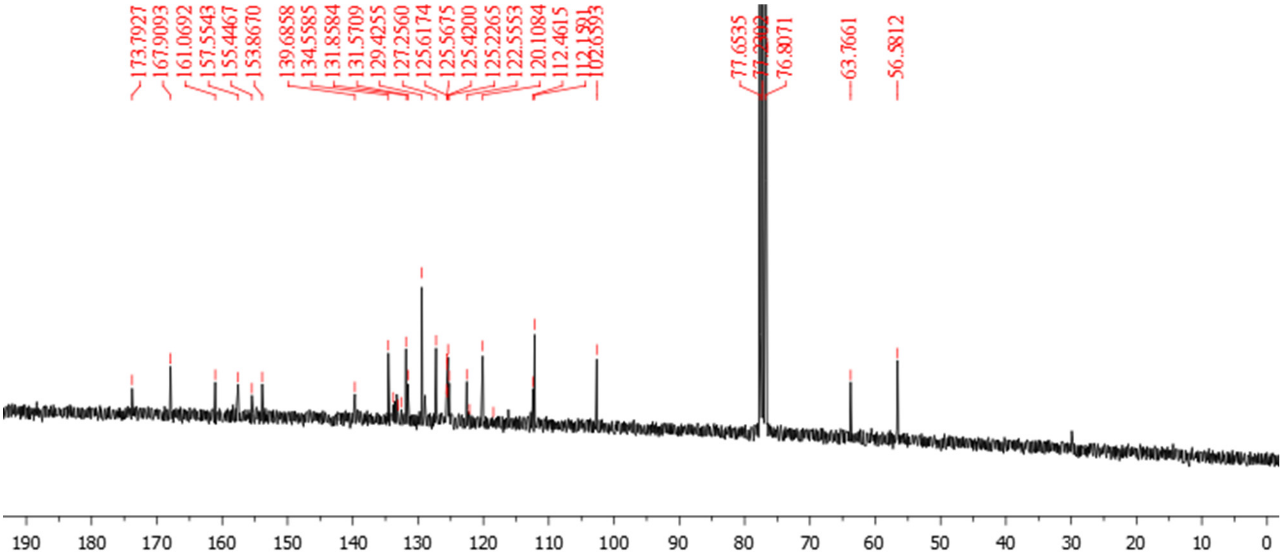

^{13}C NMR Spectrum (75 MHz, CDCl3) of 3l.

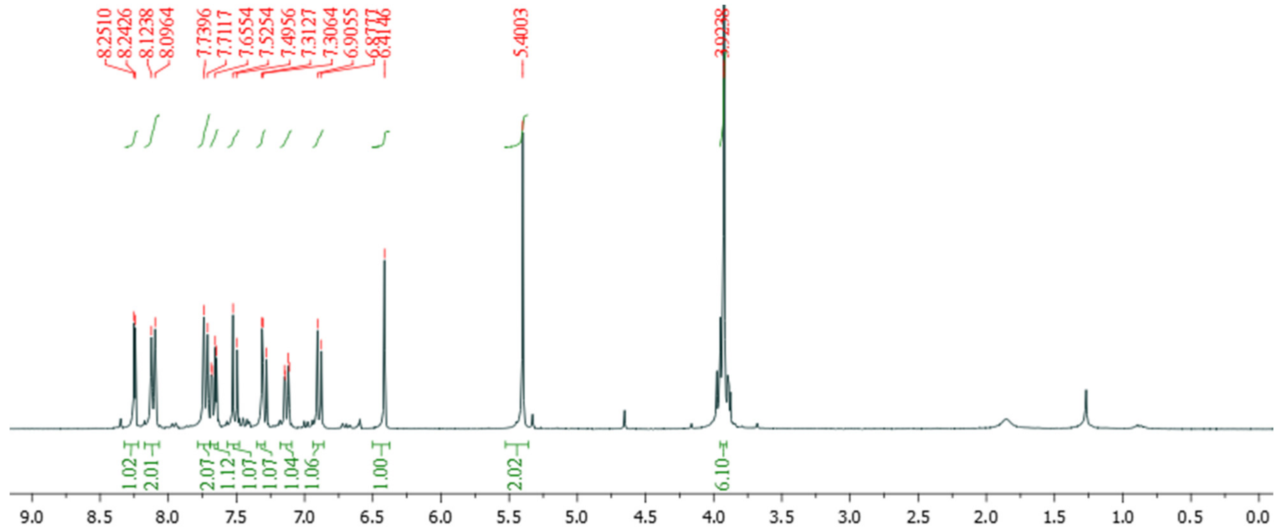

^{1}H NMR Spectrum (300 MHz, $CDCl_3$) of 3m.

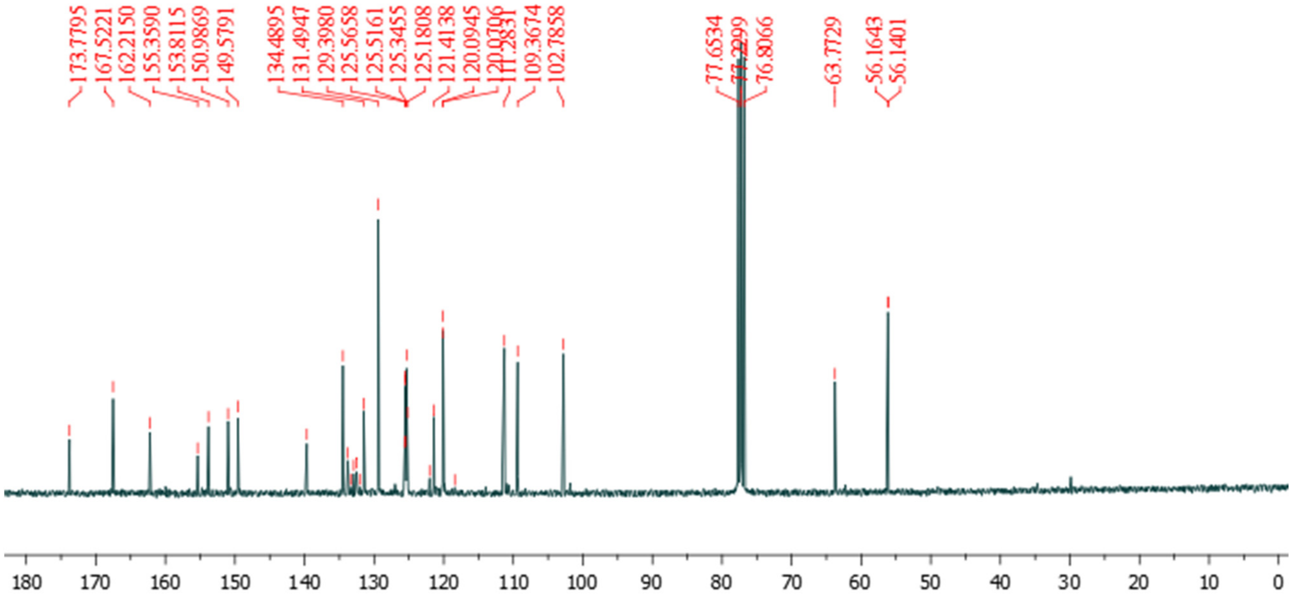

^{13}C NMR Spectrum (75 MHz, $CDCl_3$) of 3m.

Table S1. The concentration-response curves used to calculate the IC_{50} values.

Compound	Curves	log(IC_{50})
1	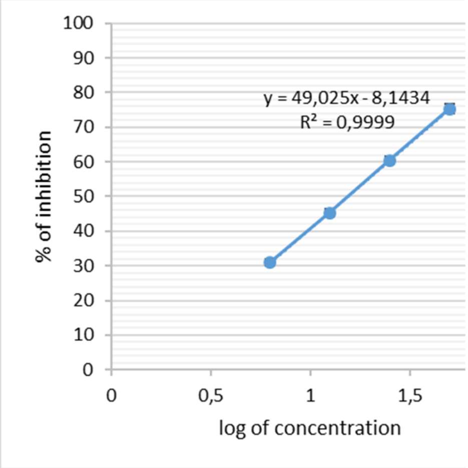	1.1859949

<table>
<tr><td>2</td><td>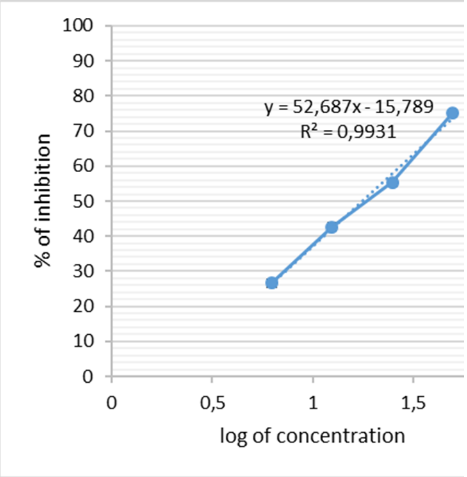
</td><td>1.24867614</td></tr>
<tr><td>3a</td><td>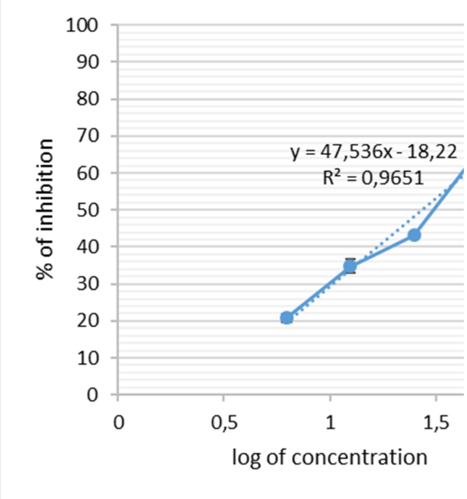
</td><td>1.43512285</td></tr>
<tr><td>3b</td><td>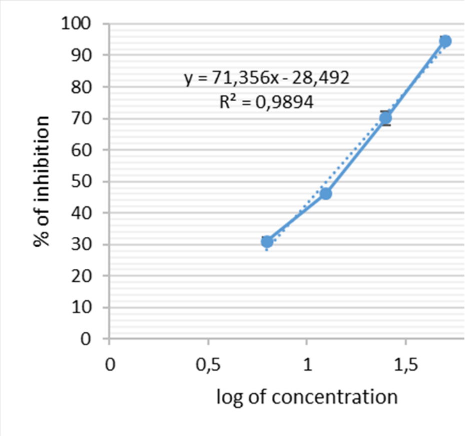
</td><td>1.10000561</td></tr>
</table>

**3c**	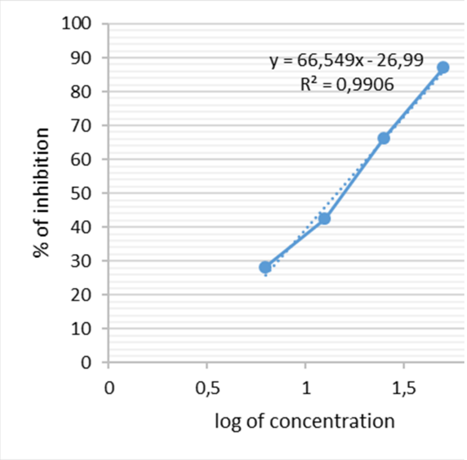	1.15689191
**3d**	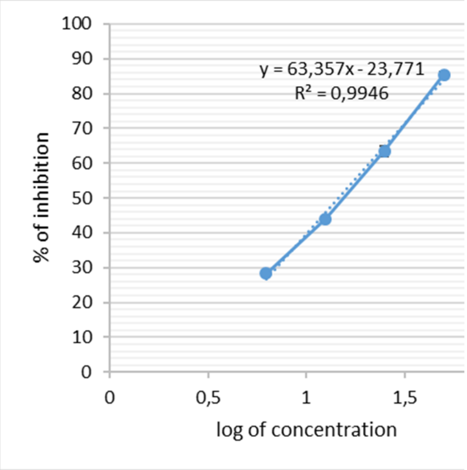	1.16437016
**3e**	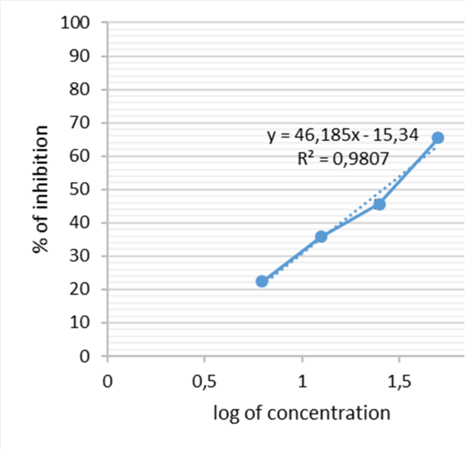	1.41474505

**3f**	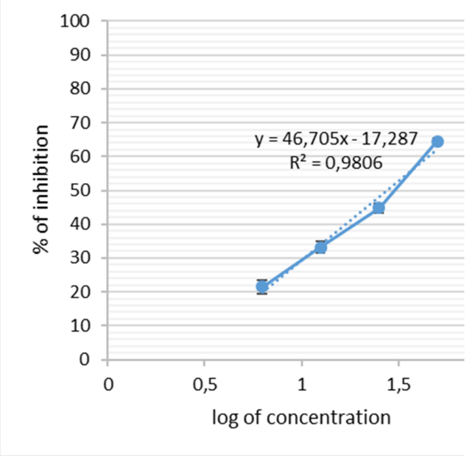	1.440680869
**3g**	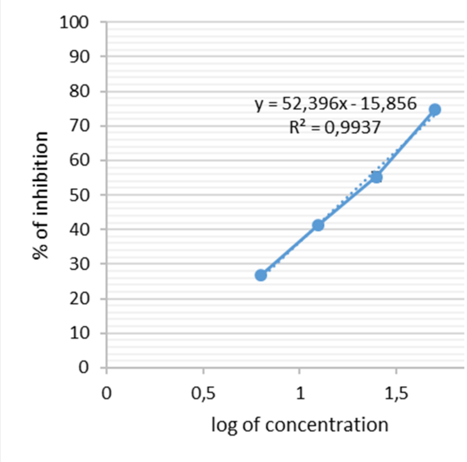	1.25688984
**3h**	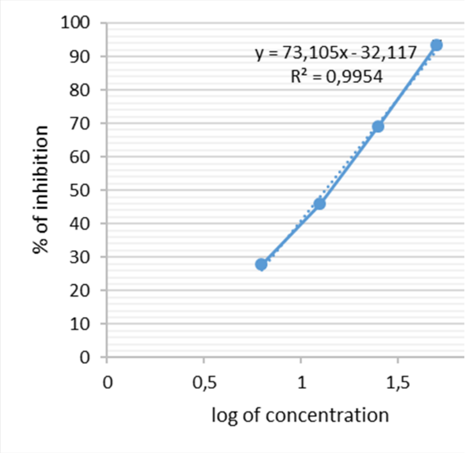	1.12327474
**3i**	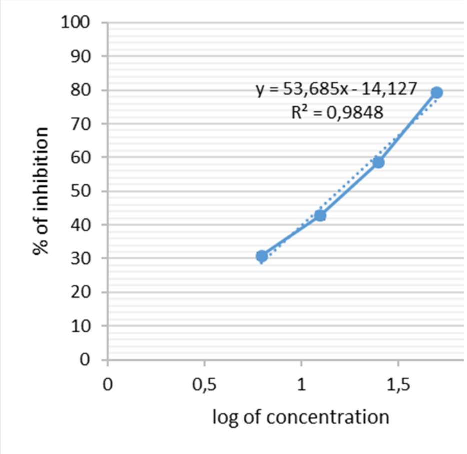	1.19450498

**3j**	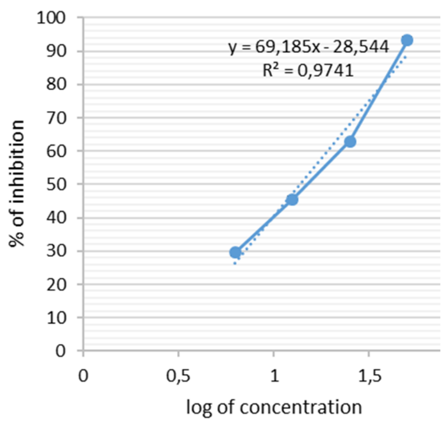	1.13527499
**3k**	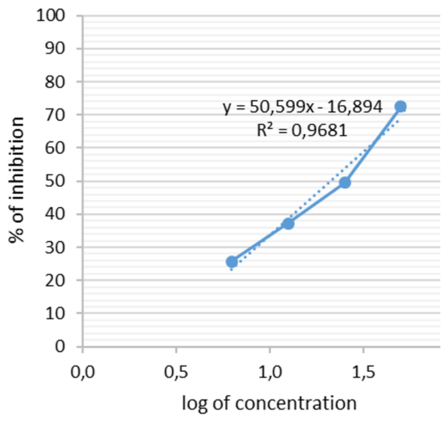	1.32204194
**3l**	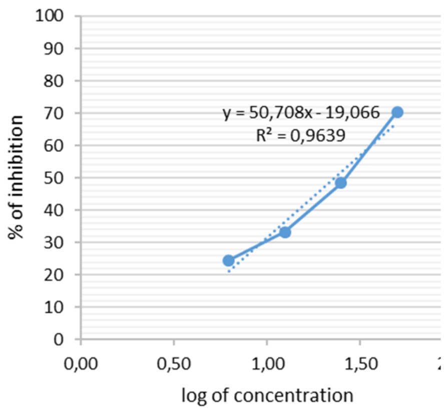	1.3620336
**3m**	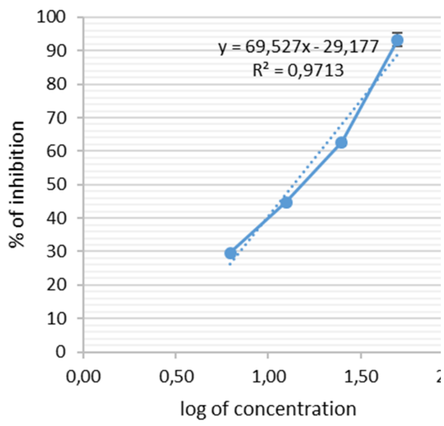	1.138795

**Acarbose** (Positive control)	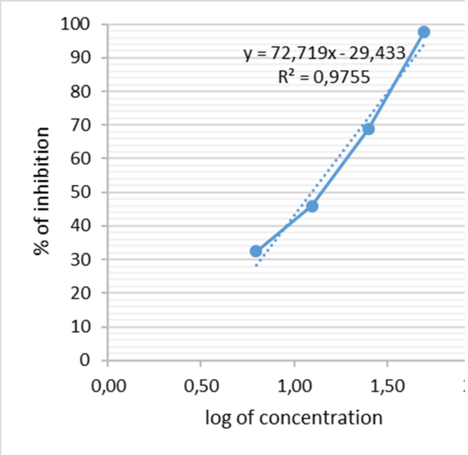	1.092328

2
